# Echocardiographic Framework for Left Atrial Appendage Morphology Assessment and Thromboembolic Risk Stratification in Atrial Fibrillation

**DOI:** 10.3390/diagnostics16193154

**Published:** 2026-09-28

**Authors:** Subramanian Jayakrishnan, Jeyakumar Anish Kumar, Nagalingam Rajendiran Shanker

**Affiliations:** 1 Department of Electrical and Electronics Engineering, Saveetha Engineering College, Chennai 602105, India; anishkumar@saveetha.ac.in; 2 Department of Computer Science Engineering, Sriram Engineering College, Chennai 602024, India; drnrshanker@sriramec.edu.in

**Keywords:** left atrial appendage, thrombus, atrial fibrillation, transoesophageal echocardiography, AgileFormer, LightGBM, thromboembolic risk, deep learning

## Abstract

**Background:** Left Atrial Appendage (LAA) causes a thrombus risk in patients with atrial fibrillation (AF). Accurate identification of LAA morphology reduces thrombotic stroke. **Methods:** This study proposes a hybrid deep learning framework, Left Atrial Appendage Thrombus Framework (LAATF), which integrates Adaptive CLAHE, optimized Chan–Vese contour extraction, and a multi-headed AgileFormer transformer to classify LAA morphology, detect thrombus, and estimate image-derived thromboembolic risk using the LightGBM-based proposed Thromboembolic Risk index (TRI) score. A dataset of 4320 temporal images from 240 patients with AF was used, comprising four LAA morphologies (chicken-wing, windsock, cactus, and cauliflower). CLAHE enhanced local contrast. The Chan–Vese model delineated appendage contours. AgileFormer extracted image-derived textural and morphological features to classify morphology and thrombus presence. LightGBM-based TRI regression estimated thromboembolic risk in AF patients with LAA. **Results:** The proposed framework achieved a morphology classification accuracy of 91–94% and thrombus detection AUC of 0.935, with a sensitivity of 93.4% and specificity of 92.6%. The computed TRI correlated strongly with actual outcomes (R^2^ = 0.98, r = 0.99). Among morphologies, cauliflower and cactus types show the highest thromboembolic risk due to their increased texture entropy and image-based features. **Conclusions:** The LAATF framework provides an interpretable, image-based technique for LAA morphology assessment and thrombus prediction.

## 1. Introduction

The Left Atrial Appendage (LAA) is a muscular, ear-shaped pouch that extends from the left atrium (LA) of the heart. The LAA is located in the pericardial space, near the free wall of the left ventricle [1]. It acts as an embryonic remnant of the LA and retains pectinate muscles, which connect to the atrial chamber. The morphology of the LAA is classified into four main types, such as (i) chicken wing, (ii) cactus, (iii) windsock, and (iv) cauliflower, based on shape and the number of lobes present [2]. Under normal sinus rhythm, the LAA performs several physiological functions, such as (i) serving as a reservoir that stores excess blood during the systole of the LA, (ii) assisting in atrial emptying through its contractile function, (iii) secreting Atrial Natriuretic Peptide (ANP) to help regulate blood pressure, and (iv) serving as a pressure buffer to reduce excessive LA pressure [3]. However, the limitation of the LAA is the formation of blood clots in individuals with atrial fibrillation (AF), which leads to strokes in these patients [4]. In patients with AF, the LAA remodels its trabeculated interior and loses contractility, increasing the risk of thrombus formation. The complex morphology of the LAA makes imaging challenging by complicating the visualization of both the LAA and any thrombus present, which necessitates the placement of a device to close the LAA [5]. Figure 1 illustrates the LAA morphology and its characteristics.

LAA morphology is detected through Transoesophageal Echocardiography (TEE), Cardiac Computed Tomography (CT), cardiac magnetic resonance imaging (CMR), 3D TEE, and Intracardiac Echocardiography (ICE). Table 1 shows the LAA detection methods and their advantages and disadvantages.

The biological relationship between the LAA and thrombus formation depends on changes in blood flow and anatomical alterations in the LAA of atrial fibrillation (AF) patients. In AF patients, atrial contraction is reduced, which leads to incomplete emptying of the LAA and remodeling of the pectinate muscles. Consequently, reduced blood flow creates conditions for blood clot or thrombus formation within the LAA. Thrombus initially develops at the tips of the LAA and spreads over the lobes and trabeculations, leading to strokes [6]. The complex shape of the LAA, with its multiple pectinate muscles, promotes thrombus formation [7]. A greater number of lobes in LAA morphology is associated with lower blood flow and an increased risk of thrombus formation. The LAA has a narrow entrance and a deep cavity, which restricts blood inflow and outflow due to acute angles and irregular bends [8]. Table 2 illustrates the relationship between LAA morphology and thrombus formation based on flow dynamics and stasis patterns. Image-derived features represent quantitative morphological measurements (lobe count, bend angles, orifice dimensions, circularity, and eccentricity) and computed texture metrics (entropy, intensity variance, and trabecular density) derived from automated image analysis of static TEE frames. These surrogate markers correlate with hemodynamic characteristics (flow pattern, stasis potential) but do not represent direct measurement of blood flow or hemodynamic parameters. Flow implications are inferred from morphometric geometry and texture characteristics, not from direct flow observation or measurement.

### 1.1. Problem Statement

The classification of the left atrial appendage (LAA) and the detection of thrombus presence are challenging tasks due to the morphological and texture feature patterns within the LAA and blood stasis. In patients with atrial fibrillation (AF), the LAA exhibits non-uniform blood movement. Slow-moving blood leads to thrombus formation, complicating diagnostic imaging. Imaging modalities have limitations in thrombus identification. (i) TEE artifacts occur due to probe alignment, and spontaneous echo contrast (SEC) mimics thrombus because of reduced blood flow patterns [9]. (ii) CT provides high-resolution anatomical details of the heart but has difficulty in identifying thrombus in low-density regions due to contrast opacification [10]. (iii) MRI shows tissue characteristics, but slow flow in the region results in false positives for thrombus due to motion artifacts [11]. The complex LAA morphologies, characterized by image-derived morphometric and textural features, are associated with increased thrombus formation risk in specific regions [12].

### 1.2. Research Gap

The imaging modality has limitations in identifying thrombus and LAA morphology due to morphological and texture feature patterns and anatomical variations among patients. These limitations reduce diagnostic consistency in differentiating true thrombus from artifacts and SEC. Existing methods fail to capture morphological and texture feature variations within the LAA, which influence thrombus formation and complicate accurate stroke risk stratification among patients with AF.

### 1.3. Research Questions

1. How does the anatomical and functional structure of the LAA influence thrombus formation in patients with AF?
2. What image-derived morphological and textural parameters accurately differentiate thrombus from artifacts and SEC in TEE?
3. How does the morphology of the LAA relate to the biological mechanisms for thrombus formation within the appendage?

### 1.4. Contributions

1. To improve the visualization of low-intensity thrombus regions through preserving morphological edges, the CLAHE image enhancement algorithm is proposed. CLAHE divides the image into regions and performs local contrast enhancement in the left atrial appendage (LAA) to identify the thrombus in low-intensity areas and enhance the edges of the LAA.
2. To accurately identify thrombus formation in the complex morphology of the LAA, such as cauliflower and cactus shapes, due to reduced velocity and multiple lobes, an intensity-based Chan–Vese contour extraction algorithm is proposed to preserve the boundaries of LAA anatomy.
3. To classify LAA morphology and the presence of thrombus, a multihead AgileFormer (AG-F) is proposed. AG-F captures global spatial relationships and contextual relationships between LAA morphology and thrombus within the LAA.
4. To estimate thromboembolic risk levels, the LAA image-based stasis is computed using image intensity, texture entropy, and shape metrics.

### 1.5. Key Novelty

The proposed LAA Morphology and Thrombus Framework (LAATF) introduces a novel hybrid methodology for enhancing LAA contours. LAATF eliminates probe-based artifacts, classifies LAA morphology through intensity-based contour extraction, and uses modern transformer-based deep learning for the extraction of quantitative, image-derived morphological and textural features from echocardiographic images to serve as surrogate markers of thromboembolic risk. Hence, the LAATF framework is designed for LAA morphology classification, thrombus detection, and thromboembolic risk prediction in patients with atrial fibrillation.

The remainder of the paper is structured as follows: Section 2 describes the techniques for detecting LAA morphology and thrombus presence, along with their advantages and disadvantages. Section 3 outlines the proposed methodology for LAA detection. Section 4 shows the results related to LAA morphology classification, the presence or absence of thrombus, and the identification of thromboembolic risk levels in stroke patients with atrial fibrillation. Section 5 concludes the paper.

## 2. Literature Survey

Table 3 summarizes the literature survey of LAA classification, thrombus detection, and stroke risk level in AF patients from different imaging modalities, techniques used, and their advantages, disadvantages, and research gaps. The direct hemodynamic measurement (e.g., flow velocity, wall shear stress, stasis time) is not feasible from static transesophageal echocardiography images. This study uses image-derived morphological and textural features as surrogate markers for the hemodynamic stasis patterns associated with thrombus formation. While these surrogate features cannot directly quantify blood flow, they capture anatomical characteristics that correlate with thromboembolic risk.

## 3. Methodology

Traditional image processing-based LAA assessment has limitations due to inter-observer variability, time-consuming manual segmentation of TEE images, and reduced accuracy due to artifacts present in the echocardiographic images. To address these issues, the LAATF framework is proposed, as illustrated in Figure 2. This framework removes artifacts such as acoustic and reverberation artifacts using Contrast Limited Adaptive Histogram Equalization (CLAHE). CLAHE enhances local contrast in small tiles to improve the visibility of lobes, orifices, thrombus, or sludge present in the LAA. Next, the Chan–Vese algorithm refines the mask to extract the LAA contour for morphology classification. Finally, the Agile Former classifies LAA morphology into categories, such as (i) Chicken Wing, (ii) Cactus, (iii) Windsock, and (iv) Cauliflower, by identifying the number of lobes present and detecting the presence or absence of thrombus through extracting textures and morphological features from the LAA in patients with AF.

Figure 3 presents a flow diagram illustrating the presence of thrombus and the early identification of thromboembolic risk using a light gradient boosting machine learning classifier (LightGBM). First, the quality of the TEE image is assessed; it contains noise from acoustic shadowing and device-related interference, which reduces clarity for analyzing thrombus and blood flow patterns. To enhance the image quality, Contrast Limited Adaptive Histogram Equalization (CLAHE) is applied. Next, the enhanced image is processed through a segmentation pipeline to extract the contours for classifying LAA morphology. The segmented contour images are fed into the AgileFormer, which extracts features such as lobe count, curvature, bending angle, appendage depth, and curvature gradients between the orifice shape and distal lobes. AgileFormer identifies the thrombus region by analyzing local intensity and irregularities in the texture pattern. Stroke risk levels are identified at an earlier stage through the LightGBM machine learning model, using morphological features of the LAA contour and thrombus texture features in AF patients.

### 3.1. Dataset Description and Annotation Protocol

The dataset contains 240 patients with AF and 4320 temporal images. Data splitting was performed at the patient level to prevent data leakage: 180 patients for training, 36 used for validation, and 24 patients for testing. No patient was included in more than one of the training, validation, and testing sets simultaneously [27,28]. The dataset comprises four distinct LAA morphological types, such as Chicken Wing, Windsock, Cactus, and Cauliflower, with each image annotated to indicate the presence or absence of thrombus. Experienced cardiologists independently assigned LAA morphology and thrombus/SEC status using TEE from M. George’s Bethlehem Ayurveda Specialty Hospital and Research Centre, Ernakulam, Kerala. Both readers were blinded to clinical data, other imaging, model outputs, and final diagnosis. A third senior cardiologist, prespecified as the adjudicator, resolved disagreements and labeled ambiguous cases.

#### 3.1.1. Reference Standard: TEE with Expert Consensus

Primary reference standard: TEE is consistent with the current practice for LAA thrombus detection. Definite thrombus: a discrete echo-dense mass with clear boundaries, persistent across gain/Doppler and views. SEC/sludge: spontaneous echo contrast (“smoke”) without a discrete mass, graded mild/moderate/severe. Possible/ambiguous: suspicious but not meeting definite thrombus criteria; sent to adjudication.

Adjudication: Consensus between the two readers was used to define the final label. Disagreement or an ambiguous case is reviewed through a third senior cardiologist.

For a subset of cases, findings were corroborated with clinical follow-up (anticoagulation response, repeat imaging, or surgery), but the final label remained anchored to TEE consensus.

#### 3.1.2. SEC vs. Thrombus at the Reference-Standard Level

Thrombus: discrete echo-dense mass, well-defined margins, persists across gain/Doppler, often in LAA tip/lobe. SEC: “smoke-like” swirling echoes, ill-defined, variable with gain and cardiac cycle, no discrete mass, often resolves with anticoagulation. SEC alone is not labeled as thrombus; ambiguous SEC + focal echo density cases were labeled as possible thrombus and adjudicated.

Table 4 summarizes the number of images in the dataset. Table 5 illustrates the dataset’s description with inclusion and exclusion criteria for TEE images and control group details corresponding to each LAA morphology type. The detailed description of the baseline architecture is provided in Supplementary File S1 along with Table S1.

For training purposes only, a class-balanced training subset is created by undersampling the majority class (chicken-wing) and oversampling the minority classes (cactus, cauliflower, windsock) to achieve exactly 1,080 images per class.

### 3.2. LAA Enhancement Using Adaptive CLAHE Enhancement

A TEE image is affected with noise due to acoustic shadowing, which reduces the accurate detection of LAA thrombus presence. To differentiate thrombus from SEC, to visualize the LAA morphology clearly, the Adaptive CLAHE Enhancement technique is proposed. Traditional CLAHE reduces the image quality in the LAA local region and thrombus boundaries due to fixed parameters of CLAHE. Therefore, to increase the image quality in the LAA boundary region, the parameters of CLAHE are selected automatically through image quality metrics, such as (i) entropy (ii) Tenengrad sharpness (iii) Root Mean Square (RMS) contrast, and (iv) noise estimate. The mathematical model of adaptive CLAHE is defined as follows:

For an input grayscale image $I\left(x,y\right)$ of intensity values in $\left[0,255\right]$, the LAA image is enhanced through local tiles $T_{i,j}$ of size t×t, and contrast amplification is limited by a clip-limit parameter $C_{L}$. The equalized output for each pixel is in Equation (1):

(1) $$I_{i,j}^{\prime }\left(x,y\right)=CLAHE\left(I\left(x,y\right);C_{L},T_{i,j}\right).$$

For every enhanced image $I_{k}^{\prime }$, image quality metrics are calculated using Equations (2)–(5). Entropy measures the information content and gray-level variability of the image in the LAA region such as lobes and flow patterns to preserve the structural pattern of LAA:

(2) $$E_{k}=-\sum_{r=v}^{255}{↓}^{r^{\prime }}\log_{2}\left(p_{r}\right)$$

where $p_{r}=\frac{h_{r}}{\sum h_{r}}$ and $h_{r}$ is the histogram count at gray level r. $p_{r}$ is the probability of gray level histogram. The edges are computed using the Sobel edge detection operator defined in Equation (3):

(3) $$T_{k}=\frac{1}{MN}\sum_{x,y}\left(G_{x}^{2}\left(x,y\right)+G_{y}^{2}\left(x,y\right)\right)$$

where $G_{x}$ and $G_{y}$ are Sobel gradients in horizontal and vertical directions. The Tenengrad sharpness is defined in Equation (3). Tenengrad defines the edges of the LAA wall, orifice, and thrombus delineation around the surrounding atrial tissue. RMS contrast enhances the separation between the blood pool, appendage wall, and thrombus.

RMS Contrast is in Equation (4):

(4) $$R_{k}=\frac{\sigma \left(I_{k}^{I}\right)}{255}$$

where $\sigma \left(\cdot \right)$ denotes the standard deviation of pixel intensities. Noise estimation enhances the visibility of lobes and the LAA region without introducing additive noise.

The noise is estimated in Equation (5):

(5) $$N_{k}=\sigma \left(I_{k}^{\prime }-\mathrm{MedianBlur}\left(I_{k}^{\prime },5\right)\right).$$

The image quality metrics do not estimate the enhancement of the image, because the image quality metrics are varied due to patient anatomy, echocardiographic settings, and acquisition settings. Hence, relative gain is used to represent how the specific quality metrics enhance the image. Relative gain is in Equation (6):

(6) $$\begin{array}{r} \Delta E_{k}=\max\left(0,E_{k}-E_{0}\right)\\ \Delta T_{k}=\max\left(0,T_{k}-T_{0}\right)\\ \Delta R_{k}=\max\left(0,R_{k}-R_{0}\right)\\ \Delta N_{k}=\max\left(0,N_{k}-N_{0}\right) \end{array}$$

where $E_{0},T_{0},R_{0},N_{0}$ are the baseline metrics of the original image.

Each gain is normalized to the range $\left[0,1\right],$ using the Equation (7):

(7) $$X_{k}^{N}=\frac{X_{k}-X_{\min}}{X_{\max}-X_{\min}+\epsilon },X\in \Delta E,\Delta T,\Delta R,\Delta N.$$

The final quality score for each CLAHE configuration is computed in Equation (8):

(8) $$S_{k}=w_{E}\Delta E_{k}^{N}+w_{T}\Delta T_{k}^{N}+w_{R}\Delta R_{k}^{N}-w_{N}\Delta N_{k}^{N}$$

where

(9) $$w_{E}+w_{T}+w_{R}+w_{N}=1,w_{E}=w_{T}=0.35,w_{R}=0.20,w_{N}=0.30.$$

The best CLAHE configuration ($C_{L}^{*},T^{*}$) is in Equation (10):

(10) $$\left(C_{L}^{*},T^{*}\right)=arg\underset{\left(C_{L},T\right)}{\max}S_{k}$$

and the corresponding enhanced image is in Equation (11):

(11) $$I^{*}=CLAHE\left(I;C_{L}^{*},T^{*}\right).$$

Figure 4 shows the LAA enhancement using Adaptive CLAHE, and Table 6 shows the image quality metrics that enhance the LAA region.

### 3.3. LAA Contour Extraction Using Optimized Chan–Vese Morphological Framework

Traditional segmentation methods, such as global thresholding and edge-based contour detection, fail to accurately delineate the Left Atrial Appendage (LAA) due to its complex anatomy and low boundary contrast. The optimized Chan–Vese model automatically extracts the LAA contour. This method integrates adaptive hybrid thresholding to generate an initial region of interest, while the Chan–Vese active contour segmentation algorithm is used for morphological refinement and precise boundary evolution. This combined approach captures both global and local intensity variations in the LAA appendage wall and its internal structures. The extracted contours are utilized for detailed morphological assessment and provide quantitative analysis of anatomical parameters, such as orifice dimension, lobe configuration, and appendage depth, which are essential for classifying LAA morphologies and assessing thromboembolic risk in patients with atrial fibrillation. The optimized-CV model is defined as follows:

To extract the initial contour, a binary segmentation of the LAA is defined in Equation (12):

(12) $$B_{g}=1\left\{I_{s}\geq t^{⋆}\right\},B_{l}=1\left\{I_{s}\leq \left(G_{\sigma_{a}}*I_{s}\right)-C\right\},B=B_{g}\vee B_{l}$$

where $t^{⋆}$ is defined as the global Otsu threshold, which separates the LAA cavity from the surrounding myocardium. $B_{l}$ represents the adaptive local threshold, which handles uneven illumination or shadowing near the appendage wall. ‘*B*’ is a hybrid mask ensuring both global and local consistency of the LAA region.

To identify the continuous edges of the LAA, morphological opening and closing operations are used to remove noise and close small gaps within the atrial cavity. The dilation operation ensures a continuous appendage structure. Hence, morphological opening and closing are defined in Equation (13):

(13) $$\mathrm{RefinedBinaryMask}\left(M_{0}\right)={\mathrm{Dilate}}^{\left(4\right)}\left(\mathrm{Open}\left(\mathrm{Close}\left(B;S_{5}\right);S_{3}\right);S_{3}\right)$$

where ‘B’ is the binary mask that contains values of 1 or 255 for the LAA region and 0 for background. $S_{5}$ is used with a structuring element of size 5 × 5. The ‘close’ operation fills the small holes in the LAA region. The ‘open’ operation preserves the overall shape by eliminating tiny pixels outside the LAA. The ’Dilate’ operation connects disjoint wall segments within the continuous region.

To refine the LAA border accurately without relying on gradient edges using CV is defined in Equation (14):

(14) $$\phi^{⋆}=arg\underset{\phi }{\min}\left[\mu \int \delta \left(\phi \right)\left|\nabla \phi \right|+\lambda_{1}\int {\left(I_{c}-c_{1}\right)}^{2}H\left(\phi \right)+\lambda_{2}\int {\left(I_{c}-c_{2}\right)}^{2}\left(1-H\left(\phi \right)\right)\right],M_{cv}=H\left(\phi^{⋆}\right)$$

where $H\left(\phi \right)$ represents the heaviside function representing the LAA interior. $c_{1}$ and $c_{2}$ show the mean intensities inside and outside the evolving contour. Hence, CV smooths irregularities in the boundary and enhances the anatomical precision of the LAA contour. Table 7 shows the LAA contour extraction process and its qualitative analysis based on orifice area, appendage depth, circularity index, and eccentricity.

Table 8 summarizes the handling of hybrid LAA morphologies during classification. Hybrid morphologies are defined as having 60% of morphological characteristics similar as another LAA type. The 60% dominant feature rule is a classification criterion used to determine whether an LAA (Left Atrial Appendage) morphology is classified as a single canonical type (chicken-wing, windsock, cactus, or cauliflower), or a hybrid morphology (mixed characteristics) [29].

During multilobe analysis, 12.3% of LAA morphologies are hybrid morphologies. Therefore, a dominant feature rule was applied to classify the LAA morphology correctly. Based on the above rule, chicken-wing morphologies are classified as 64%, cactus as 21%, windsock as 10% and cauliflower as 5% based on lobe count, bend angle, depth-to-orifice ratio, and curvature. A chicken wing has dominant appendage with minor cactus-like lobes. Hence, it is classified as chicken-wing using the above LAA morphology. Table 8 describes the handling of LAA morphology. Table 9 shows the LAA morphology with primary and secondary features and its classification based on the 60% rule.

### 3.4. LAA Morphology and Thrombus Classification Using AgileFormer (AForm)

In LAA analysis, both local texture details, such as the LAA appendage wall, thrombus edges, appendage lobes, and global morphological structures, such as Chicken Wing, Cactus, Windsock, and Cauliflower, are important. Traditional CNN models capture local pixel dependencies but fail to acquire long-range relationships. Conversely, transformer models capture global dependencies but lose boundary details. Therefore, A Form addresses these limitations through a depth-wise convolution layer and a multiheaded attention mechanism. AgileFormer utilizes depth-wise convolution layers to capture local and boundary information effectively. AgileFormer preserves the appendage geometry through multiheaded self-attention mechanism. Figure 5 shows the AgileFormer architecture for LAA morphology and thrombus classification.

The patch embedding layer of AForm is in Equation (15):

(15) $$Ts=\mathrm{LN}\left(\mathrm{Flatten}\left({Conv2D}_{7\times 7,\;s=4,\;p=3}\left(X_{s}\right)\right)\right),s\in \{c,e\}Ts$$

where the patch embedding layer converts the contour ‘c’ and the enhanced the image ‘e’ into tokens.

The depth-wise convolution layer extracts local thrombus boundaries and contour edges from the input images. It is defined in Agile Block and in Equation (16):

(16) $$F≅F+DWConv\left(F\right)⊙\sigma \left(DWConv\left(F\right)\right)$$

where the convolution layer captures the spatial dependency between lobes and the orifice. DWConv represents the depth-wise convolution.

The self-attention layer extracts the global shape dependencies among LAA lobes and wall curvature. The global feature encoding is in Equation (17):

(17) $$A=\mathrm{Softmax}\left(\frac{QK^{⊤}}{\sqrt{d_{h}}}\right),Y=AVW_{o}$$

where Q represents the query vector, which shows the lobe orientation, adjacent wall segments, and thrombus texture. ‘K’ shows the descriptive signature of a patch such as a lobe or thrombus region. ‘V’ is the actual content of the patch such as thrombus presence and LAA morphology. The Agile Block combines the local thrombus texture and global morphological features. These global encodings acquire static-frame representations of lobe orientation and texture irregularities of image derived stasis patterns. Table 10 shows the model configuration of AForm. Full implementation details of the LAATF framework are added in Supplementary File S1 with Tables S2–S4.

### 3.5. Thromboembolic Risk Index Among Patients

The thromboembolic risk index (TRI) was computed using a machine learning model trained on image-derived surrogate features, quantitative morphological measurements (orifice area, appendage depth, circularity, and eccentricity) and computed textural metrics (entropy, intensity variance) extracted from static TEE images by the AgileFormer model. These features serve as indirect indicators of thromboembolic risk and do not represent direct hemodynamic measurements (such as blood flow velocity or wall shear stress). The TRI functions as a morphology-based risk score rather than a hemodynamic quantification. The extracted features were fed through the LightGBM model to predict thrombus presence or absence among patients with AF. Thrombus-related features were excluded from the proposed TRI score estimation to avoid data leakage and the TRI risk score was derived only from only independent pre-outcome predictors. Figure 6 illustrates the important features from LAA images used to predict the risk level of patients with AF with the LightGBM model and the TRI calibration plot. SHAP waterfall plots for five representative patients indicate how TRI risk scores are constructed from morphological and textural contributions. For Patient C (TRI = 0.87), texture entropy (+0.34) and density ratio (+0.28) drive high-risk classification, while Orifice Area (−0.12) provides a protective effect. The binned calibration plot shows excellent agreement between predicted TRI probabilities and observed thrombus prevalence (Calibration Intercept: 0.02, Slope: 0.98). The model is well-calibrated across the risk spectrum.

Table 11 illustrates the comparison between LightGBM, XGBoost, and an MLP Neural Network for predicting TRI from LAA and morphological and texture features. The results in Table 11 show that LightGBM achieves higher prediction accuracy than the other classifiers because of its ability to capture nonlinear relationships between LAA morphological features, texture entropy, and flow stasis.

## 4. Results and Discussions

The classification performance of LAA morphology and the presence or absence of thrombus is analyzed using metrics such as precision, recall, F1-score, accuracy, and the AUC curve. These metrics are defined in Equations (18)–(21). Table 12 shows the unit-wise analysis of each hierarchy. Table 13 illustrates the included and excluded features for thrombus and LAA detection. Table 14 shows the performance analysis of LAA morphology classification and thrombus detection. Figure 7 shows the confusion matrix of LAA morphology and thrombus presence.

(18) $$Precision=TP/\left(TP+FP\right)$$

(19) $$Recall=TP/\left(TP+FN\right)$$

(20) $$F1\text{-}Score=2\times \left(Precision\times Recall\right)/\left(Precision+Recall\right)$$

(21) $$Accuracy=\left(TP+TN\right)/\left(TP+TN+FP+FN\right)$$

From Equations (18)–(21),

TP = True Positives-correctly identified thrombus or morphology type

TN = True Negatives-correctly identified non-thrombus or different morphology

FP = False Positives-incorrectly predicted thrombus or morphology

FN = False Negatives-missed thrombus or misclassified morphology.

Table 15 describes the difference between SEC and thrombus using the LAATF Framework based on LAA texture and boundary sharpness features. Hence, LAATF distinguishes SEC from thrombus using texture entropy ratio and density mapping. The dataset included 58 patients with thrombus-positive and 182 with SEC-only, out of 240 patients with AF. Patient-level mean feature values were used for group comparison with an independent *t*-test and a linear mixed-effects model at the patient level. All *p*-values are indicated at the patient level. Figure 8 shows the cohort-level comparisons of 240 patients with AF for thrombus detection and TRI risk stratification.

Table 16 shows the failure case analysis of the proposed framework for identifying misclassification of LAA morphology due to morphological similarity and imaging artifacts. Cactus and cauliflower morphologies are misclassified most frequently because both have multiple trabeculated lobes and irregular appendages, which affect the structural boundaries of both LAA morphologies. Therefore, most failure cases are analyzed based on anatomical similarity and echocardiographic artifacts. Table 17 shows the performance of LAATF compared with baseline methods. Table 18 describes the cross-validation-based feature selection and tuned hyperparameter optimization of the proposed model for LAA morphology and thrombus detection. Table 19 shows the ablation analysis of the proposed LAATF framework for LAA morphology classification and thrombus risk level prediction in patients with AF with thrombus presence.

The thromboembolic risk level is estimated for each patient based on LAA morphology associated with image-derived features in Table 19.

### 4.1. Image-Derived Stasis Index (IDSI)

A composite surrogate metric computed from normalized texture entropy, morphological complexity measures (circularity, eccentricity, trabecular density), and geometric parameters (depth-to-orifice ratio) derived from automated image analysis. IDSI does not represent direct measurement of blood flow or hemodynamic stasis but rather an image-derived morphological complexity score that correlates with thromboembolic risk. Values range from 0 (low complexity/low stasis risk) to 1.0 (high complexity/high stasis risk.

A total of 240 patients with AF were analyzed. Table 20 provides the descriptive correlation analysis. Thrombus presence was treated as the observed outcome and was not used in TRI construction. High inter-feature correlation describes redundancy between image-derived stasis and texture entropy. Table 21 explains the ablation study of the proposed LAATF framework.

### 4.2. Discussions

An image-derived thromboembolic risk index (TRI) was estimated as a handcrafted score from independent morphological and image-based texture features extracted from TEE images. The TRI includes image-derived stasis, texture entropy, shape complexity, circularity, and eccentricity. A LightGBM model was used as a classifier using the same independent image-derived features to estimate thrombus status or risk stratification.

The traditional system, **CHA_2_DS_2_-VASc**, estimates stroke risk using demographic data, including C—Congestive heart failure/LV dysfunction, H—Hypertension, A_2_—Age ≥75 years, D—Diabetes mellitus, S_2_—Prior stroke/TIA/thromboembolism, V—vascular disease (prior myocardial infarction, peripheral artery disease, aortic plaque), A—Age 65–74 years, and Sc—Sex category (female). However, this traditional system fails to predict stroke risk based on individual imaging biomarkers, morphological and texture features, and anatomical variations in left atrial appendage (LAA) morphology. Therefore, TRI is calculated through personalized assessments of a patient’s LAA morphology using Equation (22):

(22) $$TRI=0.40\times F_{stasis}+0.25\times T_{entropy}+0.05\times \left(1-C_{index}\right)+0.05\times G_{shape}+0.05\times E_{ccentricity}.$$

Table 22 presents the patient-specific risk stratification comparing traditional methods and the proposed TRI score method. Table 23 illustrates the performance metrics regarding the presence and absence of thrombus across various LAA morphologies, along with stroke risk stratification using both the proposed and state-of-the-art techniques.

### 4.3. Simple Cohort Design for TRI/Stroke Validation Study

Cohort

**Population:** 240 patients with atrial fibrillation (AF) undergoing TEE for LAA evaluation.

**Setting:** Single center, India, between 12 April 2024 and 12 October 2024

**Inclusion:**

Confirmed AF (paroxysmal, persistent, or permanent), TEE with adequate LAA imaging, ability to follow up for at least 6 months.

**Exclusion:** Prior stroke within 3 months before index TEE, missing key data (CHA_2_DS_2_-VASc components, TRI calculation), follow-up <6 months with no event and no censoring information.

**Baseline risk stratification**

CHA_2_DS_2_-VASc was calculated at baseline using standard criteria.

**Risk groups:**

Low: 0–1, Intermediate: 2–3, High: ≥4

TRI score (image-derived) calculated from TEE-based LAA morphology and other features.

TRI thresholds define low-, intermediate-, and high-risk TRI categories.

**Follow-up and outcome**

Follow-up period: 6 months from index TEE.

Primary endpoint: Ischemic stroke or systemic embolism within 6 months.

Event ascertainment: EMR review and outpatient visits.

Adjudication: Suspected events reviewed by 2–3 clinicians (neurologist + cardiologist), blinded to TRI.

Definite stroke = clinical stroke + imaging (CT/MRI) or specialist confirmation.

**Censoring**

Patients are followed from index TEE until the first of: Stroke/systemic embolism (event), Death (if no prior stroke), Last known follow-up (if lost or incomplete),

6 months (if no event). Patients without an event are censored at the last known contact or 6 months.

The integration of the LAATF framework into echocardiographic evaluation provides a non-invasive technique for early stroke prevention and personalized treatment planning. Accurate delineation of morphology assists interventional cardiologists in selecting the appropriate device to minimize procedural complications. Optimizing anticoagulant therapy involves adjusting the patient’s dosage as needed. The LAATF framework supports personalized stroke risk monitoring to improve patient outcomes. Detection of LAA and thrombus prediction identifies diseases such as AF, ischemic stroke, cardioembolic stroke, transient ischemic attack, heart failure, hypertension, coronary artery disease, and peripheral arterial embolism. LAA thrombus is strongly related to abnormal blood flow and clot formation. Therefore, a pragmatic and standardized echocardiographic approach improves both the detection and differential diagnosis of LAA and thrombus, and also enhances thrombus-based risk assessment and clinical decision-making [37].

### 4.4. Limitations

External validation using multi-center clinical datasets is still required to confirm the model’s generalizability. Moreover, the TRI framework is defined based on image-based morphological and texture feature parameters without incorporating broader clinical variables such as CHA_2_DS_2_-VASc components. This framework operates on static TEE frames rather than dynamic cine sequences. Consequently, it does not provide direct hemodynamic measurements, such as flow velocity, wall shear stress, or stasis time. Instead, all reported measures are image-derived surrogate features (quantitative morphological measurements and computed texture metrics) that correlate with, but do not directly quantify, the blood stasis patterns in thrombus formation. The TRI score functions as an image-based risk predictor derived from static structural characteristics, not as a direct measurement of hemodynamic parameters.

## 5. Conclusions

The proposed LAATF framework is used for automated assessment of left atrial appendage morphology and thrombus detection in patients with atrial fibrillation. By combining adaptive contrast enhancement, optimized contour segmentation, and transformer-based feature extraction, the method achieved strong classification performance, with morphology accuracy of 91–94% and thrombus detection performance with an accuracy of AUC 0.935, sensitivity of 93.4%, and specificity of 92.6%. Image-based analysis acquires clinically relevant structural patterns in the left atrial appendage and supports standardized echocardiographic evaluation. The TRI score further showed exploratory value as an image-derived risk indicator and agreement with cardiologist-assessed clinical risk within the study cohort. Image TRI, in conjunction with cardiologist TRI-p, estimates the risk for visually complex echocardiographic findings and categorizes it into a more interpretable risk profile. The proposed study was conducted on a single-center cohort of 240 patients with AF. The TRI framework was derived from image-based morphological and texture features extracted from static TEE frames without incorporating broader clinical risk variables, such as complete CHA_2_DS_2_-VASc parameters, laboratory biomarkers, cine-flow analysis, or multicenter demographic variability. The proposed framework is used as a decision-assistance tool for thromboembolic risk stratification and thrombus assessment and is useful for expert clinical interpretation.

## Figures and Tables

**Figure 1:**
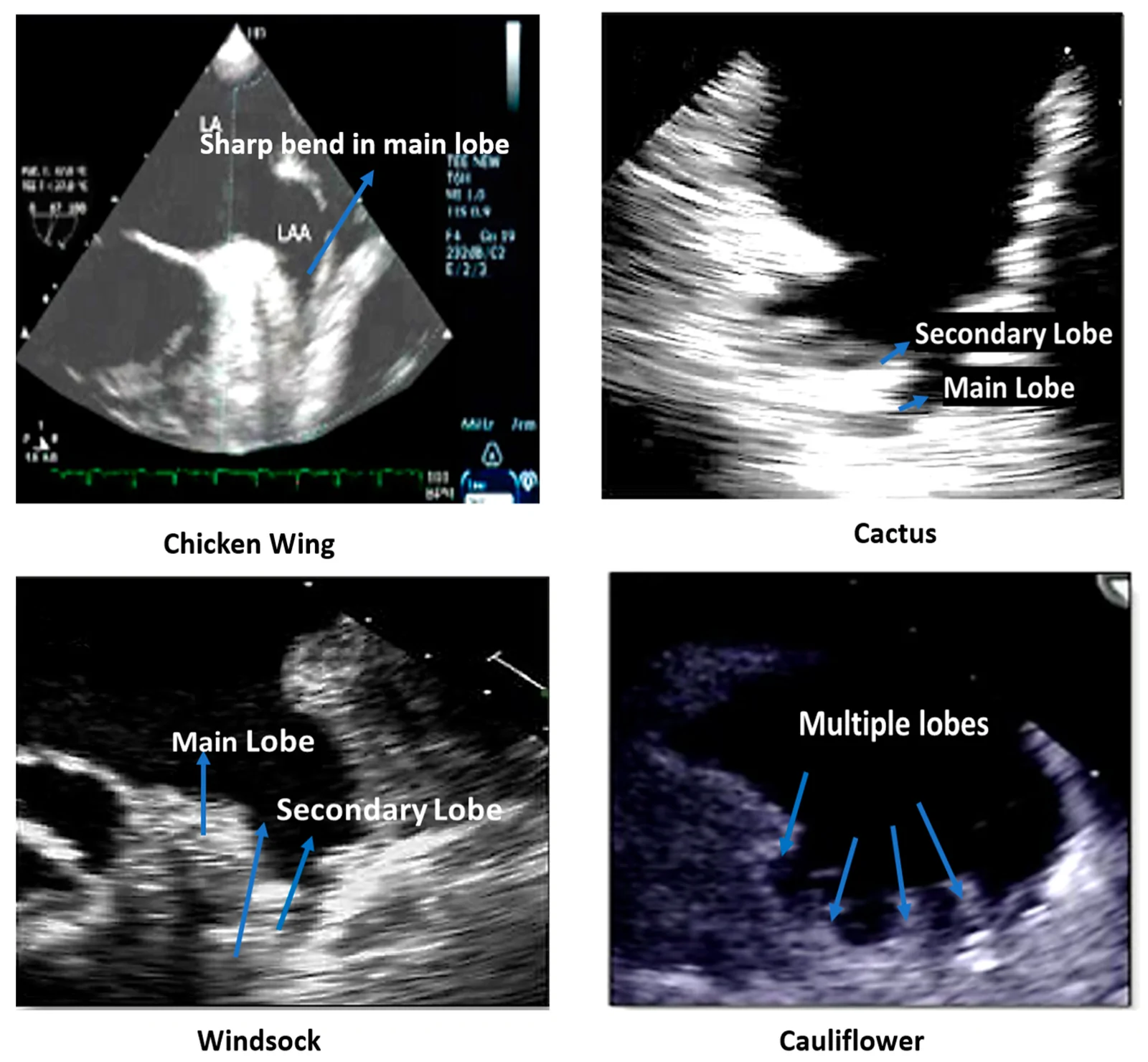
LAA morphology.

**Figure 2:**
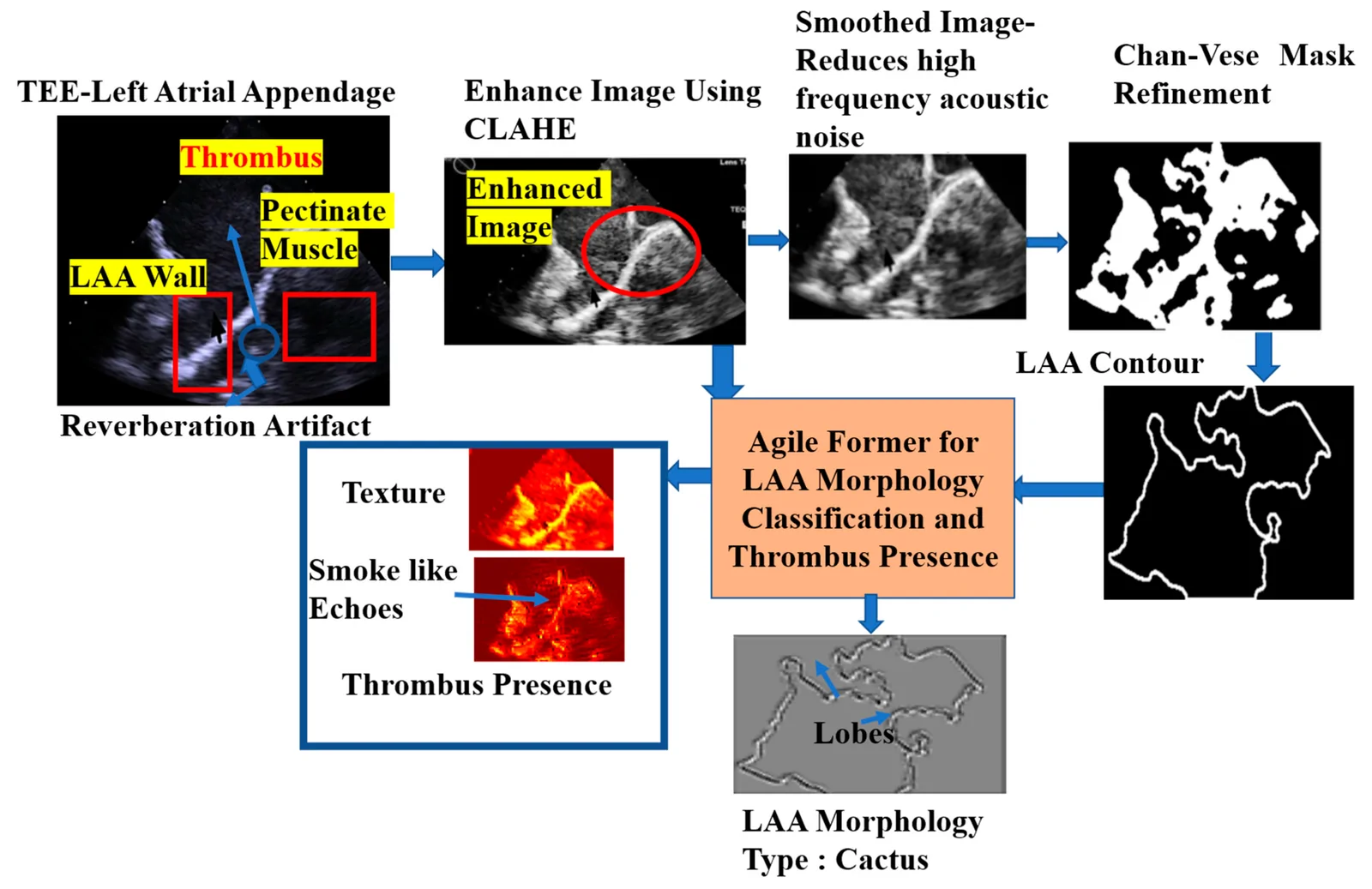
LAATF framework for classifying LAA morphology and identifying thrombus presence.

**Figure 3:**
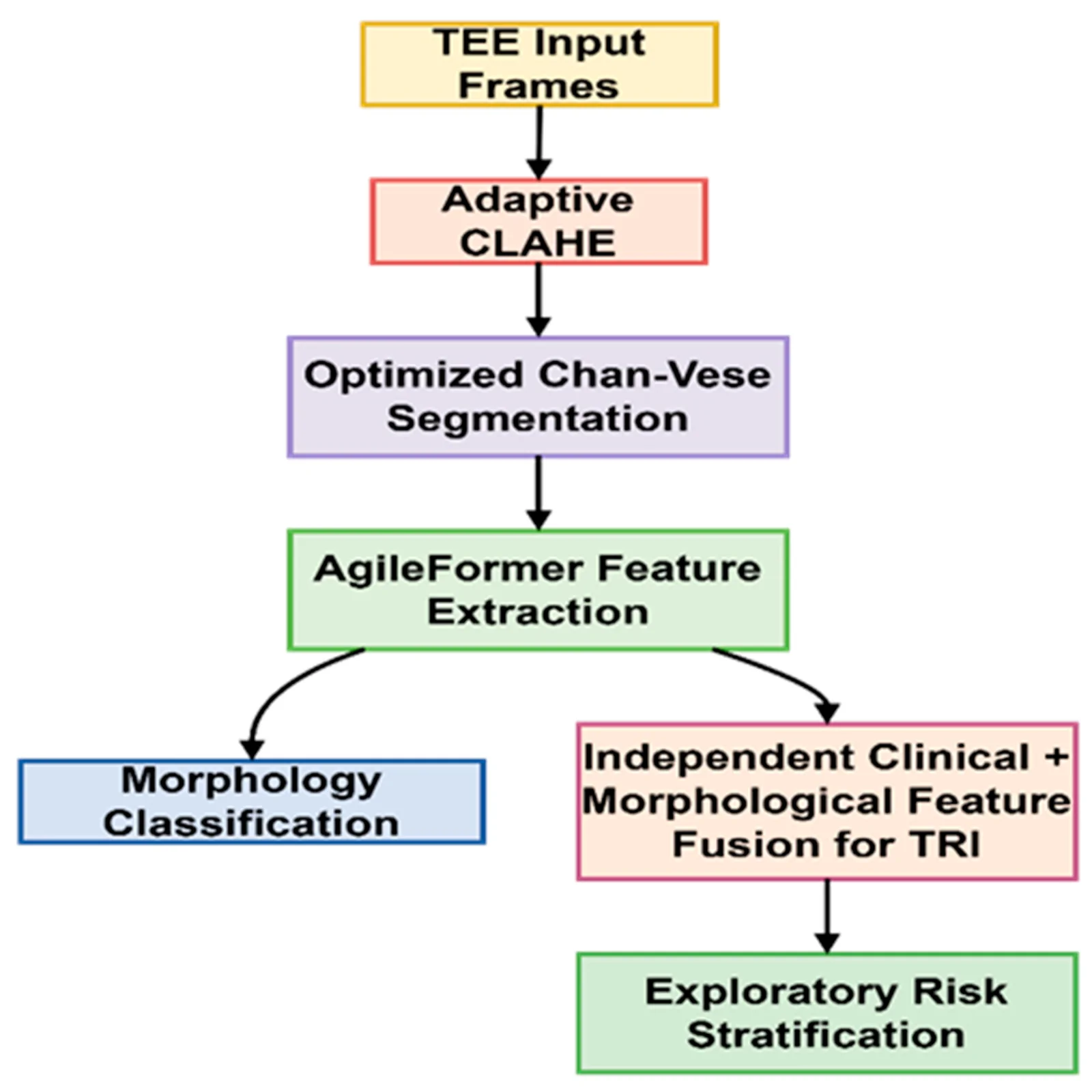
A flow diagram of thrombus presence in the LAA and thromboembolic risk identifications.

**Figure 4:**
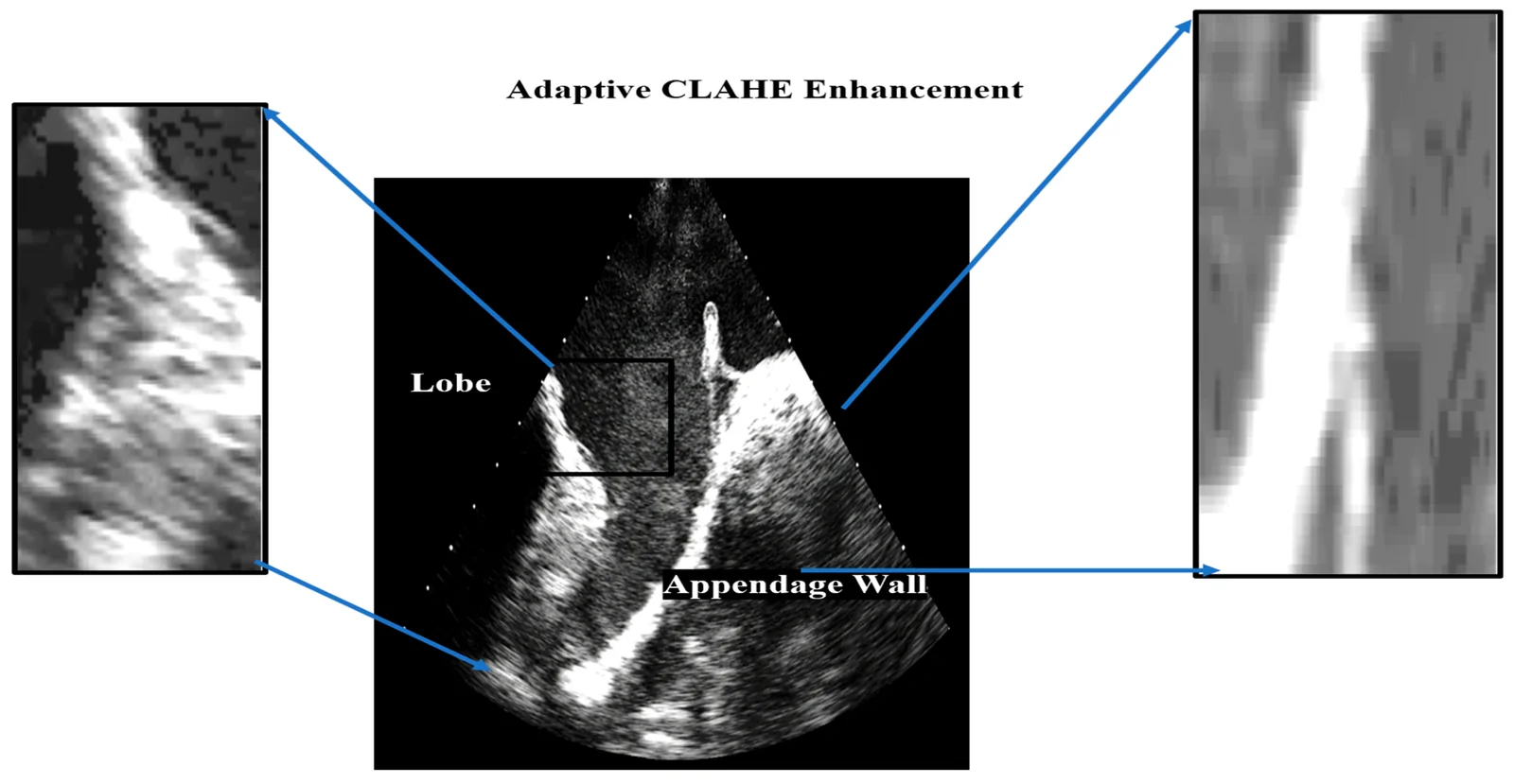
LAA enhancement using adaptive CLAHE.

**Figure 5:**
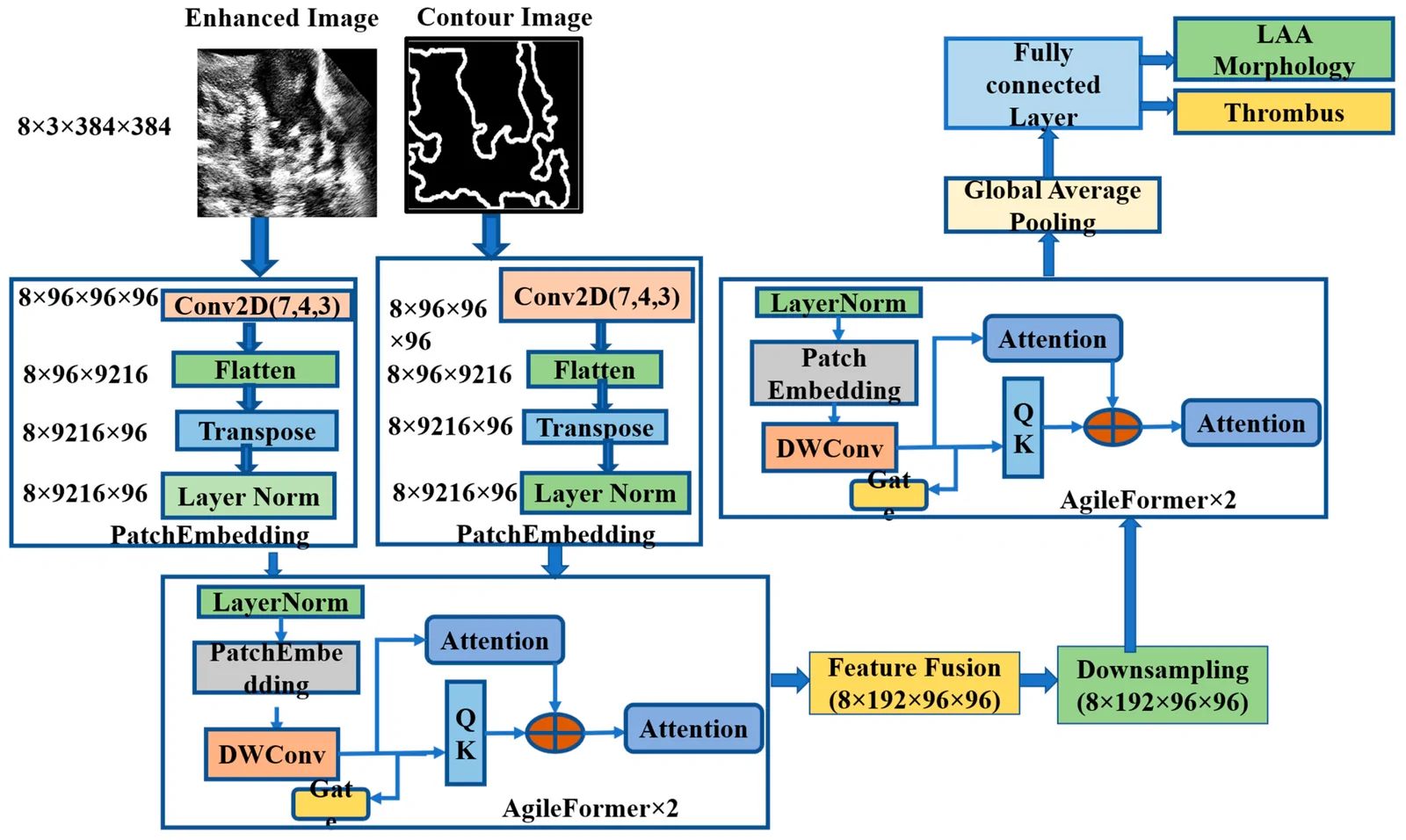
AgileFormer architecture for LAA and thrombus classification.

**Figure 6:**
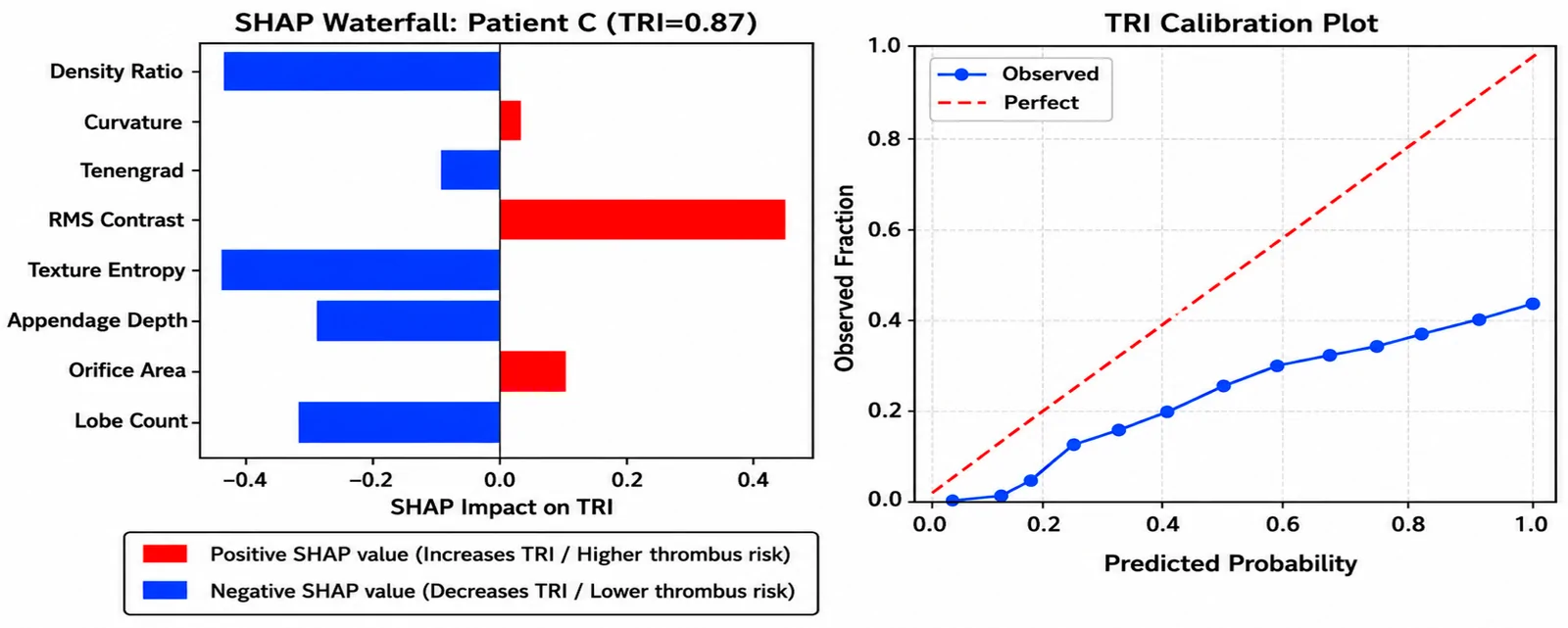
Feature importance for stroke risk prediction and TRI calibration plot.

**Figure 7:**
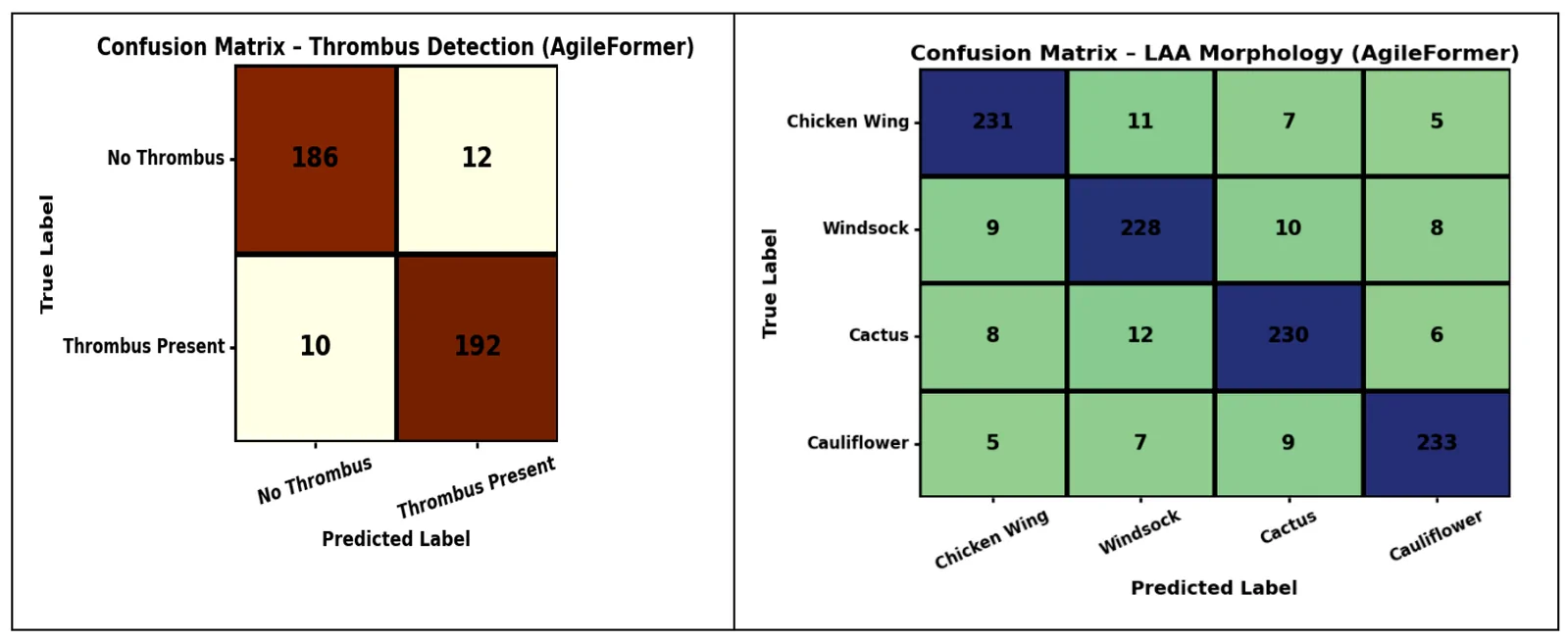
Confusion matrix for LAA morphology and thrombus presence.

**Figure 8:**
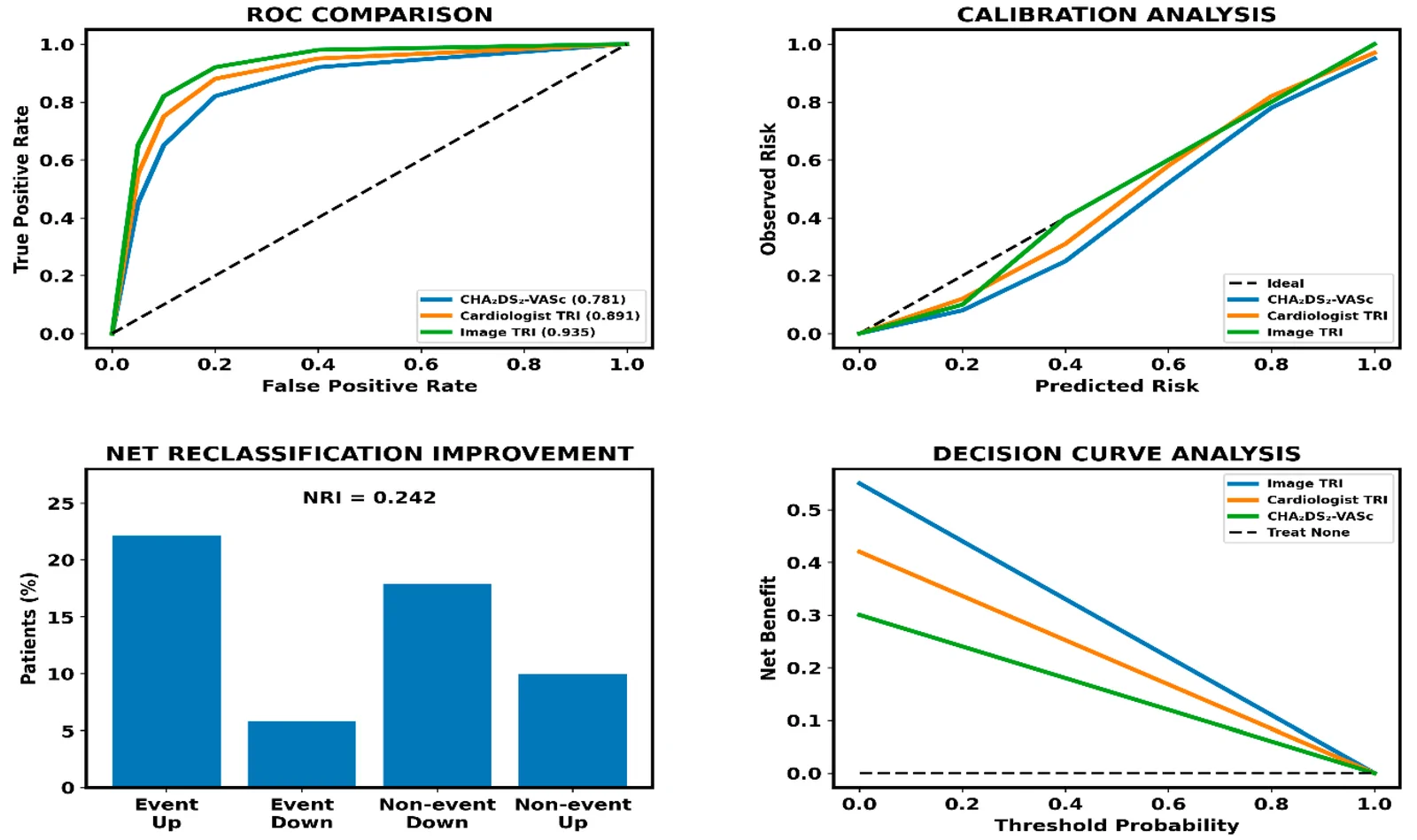
Comparative evaluation of thromboembolic risk stratification methods using ROC analysis, calibration assessment, net reclassification improvement, and decision curve analysis.

**Table 1:** LAA detection methods.

Detection Method	Methodology	Detection Technique	Advantages	Disadvantages
Transoesophageal Echocardiography (TEE)	Inserting an ultrasound probe into the esophagus to capture high-resolution images of the LAA.	Direct visualization of LAA morphology, thrombus detection.	Gold standard for thrombus detection; high spatial resolution; real-time imaging.	Semi-invasive; requires sedation; may cause esophageal discomfort or complications.
Transthoracic Echocardiography (TTE)	Non-invasive chest wall ultrasound using 2D or 3D probes.	Visualizes cardiac chambers and sometimes LAA with limited clarity.	Widely available; safe; useful for initial screening.	Limited resolution for LAA; poor visualization in obese or lung-diseased patients.
Cardiac Computed Tomography (CT)	Uses contrast-enhanced CT angiography to reconstruct 3D images of the heart and LAA.	3D reconstruction to evaluate LAA shape, volume, and presence of thrombus.	precise morphology classification; rapid scan time.	Radiation exposure; contrast nephropathy risk; cannot assess real-time flow.
Cardiac Magnetic Resonance Imaging (MRI)	Uses magnetic fields and gadolinium contrast for soft tissue characterization.	Phase-contrast or delayed-enhancement MRI for LAA visualization and thrombus detection.	Superior soft tissue contrast; no radiation	Long scan time; expensive
3D Echocardiography (3D-TEE/TTE)	Advanced echocardiographic reconstruction for full 3D morphology.	Volumetric rendering of LAA with angle-independent views.	Detailed morphology and ostium analysis	Technically demanding; high computational requirements; dependent on operator skill.
Intracardiac Echocardiography (ICE)	Catheter-based ultrasound imaging performed inside the heart chambers.	Real-time imaging of LAA from within the left atrium.	High-resolution intra-procedural guidance for LAA closure	Invasive, costly; used mainly during interventional procedures.
Contrast-Enhanced Echocardiography	Injection of ultrasound contrast to improve endocardial border delineation.	Contrast-enhanced flow visualization and cavity definition.	Enhances thrombus detection when TEE images are unclear.	Risk of allergic reaction; requires contrast administration.

**Table 2:** LAA morphology with thrombus formation.

LAA Morphology Type	Structural Description	Image-Derived Morphometric Features and Flow Implications	Nature of Thrombus Formation	Typical Thrombus Site	Thromboembolic Risk Level
Chicken Wing	Dominant lobe with sharp bend or fold resembling a bent elbow.	Single dominant lobe with sharp bend (morphometric feature; bend angle > 60°). Texture entropy 4.3 ± 0.2, circularity index 0.82 ± 0.03, minimal trabecular density. Morphometric profile suggests favorable geometry for blood evacuation.	Thrombus is small, laminated, and mural	Near the bend or distal tip of the fold.	Low
Windsock	One dominant long lobe with variable secondary lobes; tubular shape.	Elongated tubular morphology with single dominant lobe (image-derived morphometric feature). Depth-to-orifice ratio 3.0–3.5, texture entropy 5.1 ± 0.3, circularity index 0.78 ± 0.02. Morphometric geometry suggests moderate flow accommodation capacity; trabecular density indicators in distal regions associated with potential for mild stasis.	Thrombus is soft and elongated	Distal apex of main lobe.	Intermediate
Cactus	Central short stem with 2–3 or more lobes projecting in multiple directions.	Multiple lobes projecting from central axis (quantified via lobe count and angular metrics). Texture entropy 6.0 ± 0.4, circularity index 0.69 ± 0.04, eccentricity 0.62. Image-derived morphological complexity metrics suggest potential for interrupted flow between multiple lobar segments.	Thrombus forms in a lobular pattern	Between lobes and distal recesses.	High
Cauliflower	Short, complex, highly trabeculated structure with no dominant lobe.	Compact, highly trabeculated architecture with no dominant lobe (image-derived structural characteristic). Texture entropy 6.4 ± 0.5, circularity index 0.60 ± 0.54, eccentricity 0.70 (highest values indicating maximum complexity). Dense trabecular network and architectural complexity metrics (image-derived) associated with maximum stasis risk based on morphometric analysis.	Thrombus is diffuse and soft	Throughout appendage cavity.	Very High

**Table 3:** Literature survey for LAA classification and thrombus detection.

Imaging Modality	LAA Morphology and Parameters	Thrombus Detection	Risk Level	Algorithm Advantages	Algorithm Disadvantages	Research Gaps
TTE, TEE [13]	LAA size, orifice size, lobe count; peak flow velocity < 0.4 m/s	ML model integratining clinical + TTE data	High (non-valvular AF)	Interpretable Logistic Regression with high sensitivity	TEE invasive; TTE less specific; risk of overfitting	Need for large-scale non-invasive imaging datasets
3D TEE [14]	Detailed 3D segmentation of LAA lobes and shape complexity	Supports thrombus localization by segmentation	Not explicitly stated	Deep learning enhances 3D morphological delineation	High computation; anatomical variability	Lack of standardized 3D LAA morphology datasets
TEE [15]	Not detailed	ML thrombogenic milieu prediction	Non-valvular AF	ML improves prediction accuracy	Sparse morphology details; unclear model validation	affecting the reliability of thrombogenic milieu pre-diction
Multimodal: TEE, CT, MRI [16]	LAA orifice, and lobe anatomy details for device planning	Guides LAA occlusion	Clinical risk stratification	Multimodal imaging captures complementary LAA features	Cost and invasiveness; modality selection complexity	Need for optimized imaging combinations linking morphology features to thrombus risk prediction
TEE [17]	Orifice shape (oval, triangular, round), depth, wall motion	TEE is the gold standard for thrombus detection	Elevated risk patients	Detailed functional morphology assessment	Operator dependency; semi-invasive	Development of automated quantitative morphology tools incorporating thrombus risk factors to reduce subjectivity
Epicardial adipose tissue imaging [18]	Epicardial fat is linked with thrombus risk	Association via imaging biomarkers	AF patients	Novel risk factor imaging inclusion	Imaging modality details limited; unclear causal link	Elucidation of how adipose morphology influences thrombus formation and improves risk stratification
Clinical + echo data [19]	Morphology not well detailed	ML thrombus prediction	Paroxysmal AF	Early prediction with clinical data integration	Limited morphological parameters included; validation scarce	Large-scale studies incorporating detailed LAA morphological features for improved thrombus risk prediction are needed
TTE + AI [19]	Morphology details limited; clinical + echo features used	AI improves non-invasive detection	Broad AF population	Improved sensitivity with AI on TTE	Low TTE spatial resolution limits morphology assessment	Need for richer morphological feature extraction
CT and TEE [20]	LAA morphology types, orifice and lobe anatomy detailed	CT and TEE compared for thrombus detection	AF with atrial tachycardia	CT non-invasive, TEE sensitive	CT radiation; TEE semi-invasive	Standardized criteria for optimal modality use considering morphology-thrombus risk prediction are lacking
3D TEE, Cardiac CT [21]	Volume and morphology are precise for intervention planning	Guides occlusion devices	Stroke prevention patients	High resolution 3D anatomical data	High cost, technical expertise needed	Need to define specific morphology-thrombus features
Medical record data + ML [22]	LAA dimensions and clinical features are combined	ML improves thrombosis prediction	Non-valvular AF	Large dataset integration	Retrospective design; morphology features coarse	Prospective integration of detailed LAA morphological parameters for enhanced risk prediction
Echocardiography for device thrombus [23]	Post-device thrombus detection focus	Device-related thrombus diagnosis	Post-occlusion patients	Clinical management guidance	Observational limits	Morphology-thrombus interplay post-device implantation
CT [24]	Predictors of thrombogenic milieu; morphology largely unexplored	Risk assessment focus	Low-risk AF	Subtle risk factor identification	Sparse morphological data	Detailed LAA morphology integration required in low-risk thrombus prediction models
Multimodal echocardiography + ML [25,26]	Echo parameters include flow; morphology not fully detailed	ML prediction of thrombus or SEC	Non-valvular AF patients	Multi-parametric echo features improve models	Variability in echo; morphology parameters limited	Prospective validation of models with comprehensive morphological features for risk stratification
Proposed	All LAA morphology types with shape and enhanced Images	Machine Learning and Deep Learning Model	patients with AF	The LAATF framework provides an interpretable, image-based technique for LAA morphology assessment and thrombus prediction.		

**Table 4:** Number of images in the dataset.

Morphology	Original Number of Images	Number of Subjects
Chicken Wing	1620 images	64 subjects
Windsock	972 images	58 subjects
Cactus	756 images	61 subjects
Cauliflower	972 images	57 subjects
**Total**	**4320 images**	**240 subjects**

**Table 5:** LAA morphology with balanced image counts and inclusion/exclusion criteria.

LAA Morphology Type	No. of Images/ Subjects	Thrombus Status (% Positive)	Inclusion Criteria	Exclusion Criteria
Chicken Wing	1080 images/ 64 subjects	9.3%	Distinct bend resembling a folded wing with one dominant lobe.	Incomplete visualization of bend or distal lobe.
Windsock	1080 images/ 58 subjects	18.9%	Tubular elongated LAA with a single dominant lobe, visible secondary lobes, a distal tip and cavity from AF patients with mild image-based stasis.	Frames with poor resolution at the distal tip.
Cactus	1080 images/ 61 subjects	28.4%	Central body with two or more lobes extending in different directions and orifice edges.	Partial visualization of lobar projections.
Cauliflower	1080 images/ 57 subjects	43.8%	Short, compact LAA with highly trabeculated interior.	Poor boundary definition due to acoustic shadowing.

**Table 6:** Image quality metrics that enhance the LAA region.

Metric	Original	Enhanced	Relative Gain	Interpretation
Entropy	4.3649	6.5500	+2.185	Higher entropy indicates better visibility of trabeculations and finer intra-appendage structures.
Tenengrad	9378.36	15,662.82	+67.0%	Sharper edges of the LAA wall, lobes, and orifice, enhancing boundary definition and structural detail.
RMS Contrast	0.174	0.196	+13.0%	Intensity separation between the appendage cavity wall improves overall visual clarity.
Noise Std	10.389	14.178	+36.5%	A mild increase in noise shows controlled trade-off from enhancement without significant loss of diagnostic quality.

**Table 7:** LAA morphology and its qualitative analysis.

Original Image/Morphology	Adaptive CLAHE	Optimized CV Mask	LAA Contour
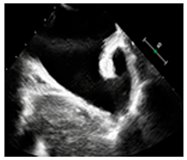 Chicken Wing	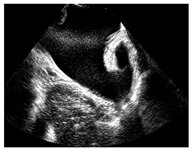	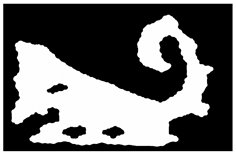	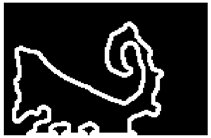 orifice area: 70 and 90 mm^2^ appendage depth is 22–25 mm, circularity index of 0.82 ± 0.03, eccentricity of 0.55. sharply curved appendage with a single dominant lobe
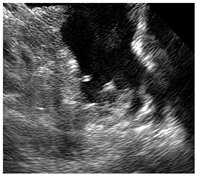 Cactus	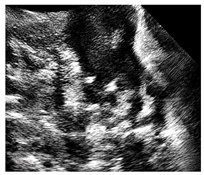	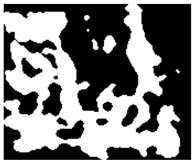	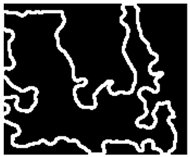 orifice area: 90–110 mm^2^ appendage depth is 24–28 mm, circularity index of 0.69 ± 0.04, eccentricity of 0.62. central body with multiple lobar projections
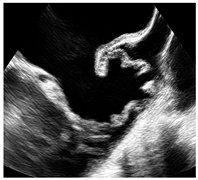 Cauliflower	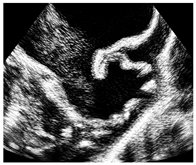	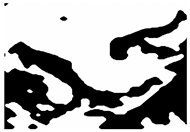	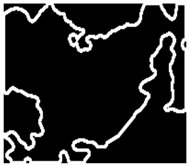 orifice area: 110–130 mm^2^ appendage depth is 18–20 mm, circularity index of 0.60 ± 0.54, eccentricity of 0.70. compact, irregular appendage with a broad orifice and minimal depth
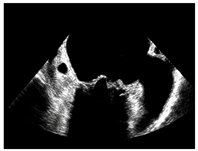 Windsock	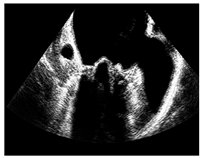	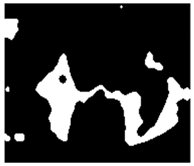	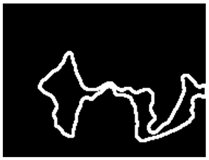 orifice area: 80–100 mm^2^ appendage depth is 30–35 mm, circularity index of 0.78 ± 0.02, eccentricity of 0.58. long, tubular appendage with a single dominant lobe

**Table 8:** Handling of hybrid LAA morphologies.

Metric	Count	Denominator	Percentage	TRI Score (Mean ± SD)	TRI-p Predicted by Expert	Layer Tuned/Used	Feature Basis
Total frames	4320	—	—	—	—	Dataset level	All TEE temporal frames
Total patients (AF)	240	—	—	—	—	Dataset level	Patient cohort
Hybrid frames (<60% match)	529	4320 frames	12.2% (12.3%)	4.87 ± 1.12	Intermediate (2–3)	AgileFormer classification layer	Mixed morphology, lobe count, bend angle, depth/orifice ratio, and curvature
Hybrid patients	29	240 patients	12.1%	4.92 ± 1.08	Intermediate (2–3)	AgileFormer classification layer	Mixed morphology across patient frames
Edge-case frames (≤40% match)	26	4320 frames	0.6%	5.14 ± 1.23	Intermediate/High	Classification layer + expert review	Weak dominant morphology, highly mixed features
Edge-case patients	14	240 patients	5.8% (corrected)	5.21 ± 1.19	Intermediate/High	Classification layer + expert review	Ambiguous dominant morphology
Reclassified hybrid frames	87	529 hybrid frames	16.4%	4.63 ± 0.98	Low/Intermediate	Hybrid decision layer	Dominant feature rule (>60%)
Reclassified hybrid patients	49	29 hybrid patients	16.9%	4.58 ± 0.95	Low/Intermediate	Hybrid decision layer	Dominant feature rule (>60%)
Canonical frames (non-hybrid)	3791	4320 frames	87.8%	3.94 ± 1.34	Low (0–1)	Morphology classification layer	Single canonical morphology
Canonical patients (non-hybrid)	211	240 patients	87.9%	3.89 ± 1.31	Low (0–1)	Morphology classification layer	Single canonical morphology
Test set accuracy (per-class)	—	48 patients	91–93%	—	—	Model evaluation layer	Patient-level classification performance
Inter-observer agreement (κ)	—	240 patients	κ = 0.87	—	—	Expert agreement layer	Cardiologist comparison

**Table 9:** Sample of LAA morphology.

Case ID	Primary Features	Secondary Features	Final Classification	Confidence	Identified Morphology
P045-F12	Single dominant lobe, sharp bend (68°)	Two minor lobes	Chicken-wing Cactus	0.87	Bend angle > 60% Chicken-wing
P128-F23	Multi-directional lobes (3)	Elongated central stem	Cactus	0.79	Lobe count dominance (3 vs. 1)
P203-F08	Long tubular shape	Bulbous distal tip	Windsock	0.92	Depth/orifice ratio > 3.5
P167-F15	Broad orifice, minimal depth	Trabeculated	Cauliflower	0.71	Orifice area > 110 mm^2^

**Table 10:** Model configuration of AgileFormer.

Layer	Configuration	Input → Output Shape	Parameters
Input Images	Dual Input Streams: Contour Image ((X_c)) and Enhanced Image ((X_e))	(3, 384, 384) → (3, 384, 384)	
Patch Embedding (per stream)	Conv2D (7 × 7, stride = 4, padding = 3) + LayerNorm	(3, 384, 384) → (96, 96, 96)	3 × 96 × 7 × 7 = 14,112
Stage 1—Agile Blocks (Local + Global)	2 × Agile Blocks (DWConv + Multi-Head Attention) per stream	(*N* = 9216, 96) → (*N* = 9216, 96)	~1.8 M (shared)
Feature Fusion (Dual-Stream Merge)	Concatenation + Conv2D (1 × 1)	(2 × 96, 96, 96) → (192, 96, 96)	2 × 96 × 192 = 36,864
Downsampling Convolution	Conv2D (3 × 3, stride = 2, padding = 1) + LayerNorm	(192, 96, 96) → (192, 48, 48)	192 × 192 × 3 × 3 = 331,776
Stage 2—Agile Blocks (Global Reasoning)	2 × Agile Blocks, heads = 6	(*N* = 2304, 192) → (*N* = 2304, 192)	~2.6 M
Global Average Pooling	Mean across all tokens	(*N* = 2304, 192) → (192)	—
Shared Fully Connected Layer	Linear (192 → 512) + SiLU + Dropout (0.2)	(192) → (512)	192 × 512 = 98,304
Morphology Classification Head	Linear (512 → 4) + Softmax	(512) → (4)	512 × 4 = 2048
Thrombus Classification Head	Linear (512 → 1) + Sigmoid	(512) → (1)	512 × 1 = 512

**Table 11:** Reason for choosing LightGBM.

Metric	LightGBM	XGBoost	MLP Neural Network	Interpretation
R^2^ Score	0.98 (0.97–0.99)	0.95 (0.93–0.97)	0.91 (0.88–0.94)	Highest predictive correlation with thromboembolic risk.
RMSE	0.023	0.025 (+8.7%)	0.032 (+39%)	Lowest prediction error.
MAE	0.018	0.021 (+17%)	0.027 (+50%)	More accurate patient-specific TRI estimation.

**Table 12:** Unit wise analysis of each hierarchy.

Task	Unit of Analysis	Aggregation Rule
Morphology Classification	Patient-level	Majority voting (mode) across all available frames
Thrombus Detection	Frame-level	Frame-wise output; positive if any single frame is “Positive”
TRI Risk Stratification	Patient-level	Mean score across all frames (post-outlier rejection)

**Table 13:** Included/excluded features of LAA and thrombus.

Feature Type	Feature	Inclusion Status	Reason	Role in Thrombus Presence/Absence
Texture Feature	Texture Entropy	Included	Represents trabecular complexity and heterogeneity	Higher entropy indicates thrombus-prone stasis
Intensity Feature	Density Ratio	Included	Reflects low-flow appendage intensity characteristics	Distinguishes normal flow from thrombus-risk regions
Morphological Feature	Orifice Area	Included	Independent anatomical biomarker	Abnormal geometry increases thrombus susceptibility
Morphological Feature	Appendage Depth	Included	Measures elongation and stagnation tendency	Deep cavities favor clot formation
Shape Feature	Circularity Index	Included	Quantifies appendage irregularity	Irregular morphology associated with thrombogenic remodeling
Shape Feature	Eccentricity	Included	Describes elongation-related geometry	Disturbed flow increases thrombus tendency
Morphological Feature	Lobe Count	Included	Indicates appendage complexity	Multiple lobes increase recirculation and clot risk
Morphological Feature	Bend Angle	Included	Characterizes LAA structural morphology	Certain morphologies correlate with thrombus risk
Flow Feature	Depth-to-Orifice Ratio	Included	Estimates stagnation-prone geometry	Higher ratio indicates increased clot probability
Flow/Stasis Feature	Image-derived Stasis	Included	Represents indirect hemodynamic stagnation	Identifies low-flow regions linked with thrombus development
Texture Feature	Intensity Variance	Excluded	Directly reflects organized clot texture	High variance strongly indicates thrombus presence
Boundary Feature	Tenengrad Sharpness	Excluded	Detects sharp thrombus edges	Sharp boundaries confirm formed thrombus
Direct Thrombus Feature	Echogenic Thrombus Core	Excluded	Explicit clot-defining feature	Direct evidence of thrombus presence
Direct Thrombus Feature	Contrast-poor Appendage Region	Excluded	Indicates thrombus-filled region	Suggests thrombus occupancy
Direct Thrombus Feature	Echo-dense Mass	Excluded	Gold-standard thrombus indicator	Confirms definite thrombus
Direct Thrombus Feature	Persistent Thrombus Boundary	Excluded	Stable clot visualization across views	Indicates existing thrombus
SEC-related Feature	Smoke-like Dynamic Echoes	Excluded	Represents SEC/thrombus manifestation	Associated with thrombus-prone state
Texture Feature	Speckle Irregularities	Excluded	Reflects clot/SEC-specific texture dynamics	Linked with thrombus appearance rather than predisposition

**Table 14:** Performance analysis of AgileFormer in LAA classification and thrombus detection.

Category	Precision (%)	Recall (%)	F1-Score (%)	Accuracy (%)	AUC (%)	Dominant AgileFormer Layer(s)	Anatomical/Textural Regions Identified
Chicken Wing	92.1	91.3	91.7	93.4	93.0	Layers 1–2 (Early Encoder)	LAA orifice contour, basal appendage bend, and proximal wall margins.
Windsock	90.8	91.9	91.3	92.7	91.8	Layers 3–4 (Mid Encoder)	Elongated appendage body, distal lobe tapering, and cavity flow axis.
Cactus	91.5	92.4	91.9	93.1	92.8	Layers 4–5 (Intermediate Encoder)	Multi-lobed appendage branches, trabecular junctions, and lobar separation lines.
Cauliflower	92.8	93.2	93.0	94.0	93.6	Layers 5–6 (Deep Encoder)	Dense trabecular network, compact appendage cavity, and irregular endocardial texture.
Thrombus	93.4	92.6	93.0	93.8	93.5	Layers 6–8 (Late Encoder and Aggregation)	Echogenic thrombus core, and contrast-poor appendage regions.

**Table 15:** SEC and thrombus differentiation using LAATF framework.

Feature	True Thrombus (*n* = 58 Patients)	SEC (*n* = 182 Patients)	*p*-Value (*t*-Test)	*p*-Value (Mixed-Effects)	ICC	Discriminative Power
Texture Entropy	6.2 ± 1.1	4.3 ± 0.8	*p* < 0.001	*p* < 0.001	0.73	Primary discriminator
Intensity Variance	12.4 ± 2.3	8.1 ± 1.5	*p* < 0.001	*p* < 0.001	0.68	High variance = organized clot
Boundary Sharpness (Tenengrad)	156 ± 23	89 ± 14	*p* < 0.001	*p* < 0.001	0.71	Sharp edges = thrombus
Speckle Pattern irregularities	Minimal (stasis)	Smoke	Visual	Visual	N/A	SEC shows dynamic patterns
Density Ratio (vs. myocardium)	0.67 ± 0.09	0.92 ± 0.07	*p* < 0.001	*p* < 0.001	0.65	Thrombus darker than myocardium

**Table 16:** Failure case analysis.

Error Type	Cases	Impact	Root Cause	Mitigation
Cactus ↔ Cauliflower	87	F1: 0.92 → 0.89	Similar trabeculated multi-lobes	Lobe separation metric
Chicken Wing ↔ Windsock	54	F1: 0.93 → 0.91	Mild bend angles	Bend angle threshold tuning
Small thrombus FN (<3 mm)	31	Sensitivity: 93.4 → 89.2%	Acoustic shadowing	Multi-frame fusion
Dense SEC FP	22	Specificity: 92.6 → 90.1%	Texture similarity	Entropy ratio threshold
Poor image quality	12	AUC: 0.935 → 0.912	Reverberation artifacts	Image quality gating

**Table 17:** Performance of LAATF compared with baseline methods.

Metric	LAATF Value	95% CI	Test vs. Baseline
Morphology Accuracy	92.3%	90.1–94.5%	vs. TransUNet (88.1%)
Thrombus AUC	0.935	0.912–0.958	vs. SegFormer (0.901)
Sensitivity	93.4%	90.2–96.1%	vs. Clinical (82.3%)
Specificity	92.6%	89.3–95.4%	vs. Clinical (79.8%)
TRI R^2^	0.98	0.97–0.99	vs. XGBoost (0.95)
TRI RMSE	0.023	0.019–0.027	vs. XGBoost (0.025)

**Table 18:** Cross-validation-based feature selection and tuned hyperparameter optimization of the proposed Model.

Fold	Feature Selection Method	Selected Features	Tuned Hyperparameters	Best Tuned Values	Morphology Accuracy (%)	Thrombus AUC	TRI ($R^{2}$)
5-Fold CV	Correlation + RFE	Entropy, Density Ratio, Orifice Area, Appendage Depth	Learning Rate, Max Depth, Estimators, Dropout, Attention Heads	LR = 0.001, Depth = 6, Estimators = 200, Dropout = 0.20, Heads = 6	92.3 ± 0.5	0.935 ± 0.006	0.98 ± 0.01
10-Fold CV	Hybrid Morphology + Texture Selection	Entropy, Circularity, Eccentricity, Curvature Gradient	Learning Rate, Embedding Dimension, Transformer Layers, Feature Fraction	LR = 0.0005, Embedding = 192, Layers = 8, Feature Fraction = 0.78	92.7 ± 0.4	0.941 ± 0.005	0.98 ± 0.01
15-Fold CV	Full Feature Fusion	Morphology + Texture + Shape + Stasis Features	Learning Rate, Estimators, Dropout, Attention Heads, Max Depth	LR = 0.0003, Estimators = 300, Dropout = 0.15, Heads = 8, Depth = 8	93.0 ± 0.3	0.946 ± 0.004	0.99 ± 0.01

**Table 19:** LAA morphology-associated image-derived features used for TRI estimation.

LAA Morphology	No. of Patients	Image-Derived Stasis Inex (IDSI)	Texture Entropy (Mean ± SD)	TRI Category Derived from Independent Features
Chicken Wing	64	0.28 ± 0.04	4.3 ± 0.2	Low
Windsock	58	0.45 ± 0.06	5.1 ± 0.3	Moderate
Cactus	61	0.61 ± 0.08	6.0 ± 0.4	High
Cauliflower	57	0.72 ± 0.09	6.4 ± 0.5	Very High

**Table 20:** Correlation analysis between image-derived stasis and observed thrombus-related features.

Parameter Pair	Pearson r	*p*-Value	Relationship
Image-derived stasis vs. Thrombus Presence	0.91	<0.001	Strong Positive
Texture Entropy vs. Thrombus Presence	0.88	<0.001	Positive
Image derived stasis vs. Texture Entropy	0.85	<0.001	Highly correlated

**Table 21:** Ablation study of proposed LAATF framework.

Model Variant	Processing Steps Included	Inputs Fed to LightGBM	MAE	R^2^ Score	Correlation (r)	Interpretation
Model-1: Baseline	Raw TEE frame → handcrafted summary descriptors	Raw-frame descriptors only	0.091	0.84	0.90	Tests whether unprocessed TEE contains usable signal
Model-2: + CLAHE	Raw TEE → CLAHE → same descriptors	CLAHE-derived descriptors only	0.072	0.89	0.93	Tests whether contrast enhancement improves the same feature set
Model-3: + Segmentation	Raw TEE → CLAHE → Chan-Vese ROI → same descriptors	ROI-based descriptors only	0.057	0.92	0.95	Tests whether segmentation adds value beyond enhancement
Model-4: + AgileFormer	CLAHE → Segmentation → AgileFormer embeddings	AgileFormer deep features	0.041	0.95	0.97	Tests whether learned representations outperform handcrafted descriptors
Model-5: + Thrombus/Flow	AgileFormer embeddings + thrombus probability + flow indices	Deep + hemodynamic features	0.032	0.97	0.98	Tests whether adding functional risk features improves TRI
Model-6: Full framework	CLAHE + Segmentation + AgileFormer + morphology + thrombus + flow	All fused features	0.026	0.98	0.99	Tests the fully integrated hybrid hypothesis

**Table 22:** Patient-specific stroke risk stratification between the traditional and proposed TRI score methods.

Patient ID	CHA_2_DS_2_-VASc [30,31] (Clinical Standard Score Method)	CHA_2_DS_2_-VASc Risk (Clinical Standard Score Method)	Cardiologist-1 TRI-p (Clinical Standard Score Method) [32]	Cardiologist-2 TRI-p (Clinical Standard Score Method)	Image TRI (Proposed Score Method)	Agreement	6-Month Stroke	Correct Risk Prediction?
P001	1	Low	0.26	0.29	0.28	Cardiologists’ vs. Image	No	Low → No stroke
P027	2	Intermediate	0.41	0.44	0.42	Cardiologists’ vs. Image	Yes	Moderate → Stroke
P056	4	High	0.60	0.63	0.61	Cardiologists’ vs. Image	Yes	High → Stroke
P108	5	High	0.77	0.81	0.79	Cardiologists’ vs. Image	No	Very High → No stroke

**Table 23:** Comparison of state-of-the-art techniques with the proposed method.

Reference (Year)	Methodology	Statistical Measure	Interpretation
Liu and Li (2024) [33]	Prospective TEE study in AF patients (*n* = 212) evaluating LAA morphology types (chicken-wing, windsock, cactus, cauliflower) and LAA emptying velocity (LAAEV).	Logistic regression: OR = 3.12 (95% CI 1.58–6.22, *p* < 0.01) for non-chicken-wing predicting thrombus; ROC for LAAEV < 20 cm/s: AUC = 0.87, Sensitivity 91%, Specificity 84%.	Non-chicken-wing morphologies and low LAAEV markedly raise thrombus risk; joint structural-functional assessment improves predictive power.
Dudzińska-Szczerba et al. (2022) [12]	Observational TEE cohort (*n* = 186) analyzing LAA shape, orifice area, velocity vs. prior stroke.	Logistic regression: OR = 2.95 (95% CI 1.42–5.87, *p* < 0.01) for non-chicken-wing; correlation LAAEV–stroke r = –0.52 (*p* < 0.001).	Complex (non-chicken-wing) morphologies and reduced velocity independently predict stroke; supports integrating LAA function into risk scores.
Cui et al. (2024) [34]	Retrospective registry (*n* = 420 NVAF) using TEE classification of thrombus/sludge depth and shape.	Thrombus frequency: 21.4% (non-chicken-wing) vs. 8.9% (chicken-wing); logistic regression OR = 2.47, *p* = 0.004.	Deep, multilobed LAA anatomy strongly correlates with thrombus/sludge formation and embolic potential.
Fang et al. (2021) [35]	CT/MRI-based CFD modelling (*n* = 60 AF) assessing wall shear stress (WSS) and stasis time.	Mean WSS: 0.41 ± 0.15 Pa (thrombus +) vs. 0.86 ± 0.28 Pa (thrombus −); stasis time ↑ 45% (*p* < 0.001).	Low WSS and prolonged stasis promote thrombus; LAA orientation dictates intra-appendage flow dynamics.
Hautmann et al. (2023) [36]	Real-world imaging cohort (*n* = 1216 AF) tracking LAA thrombus resolution and outcomes under anticoagulation.	Kaplan–Meier: HR = 2.68 (95% CI 1.74–4.09, *p* < 0.001) for stroke/death in thrombus +; thrombus-resolution HR = 0.46 (*p* = 0.008 *).	Persistent thrombus predicts poor outcomes; resolution lowers risk—underscoring importance of morphology-guided therapy.
Proposed (2025): AgileFormer + LightGBM (LAATF)	TEE frames (*n* = 4320) → CLAHE → LAA segmentation → AgileFormer texture and flow feature extraction → LightGBM regression to compute TRS.	Morphology classification accuracy = 91–94% (Chicken-wing 93.4, Windsock 92.7, Cactus 93.1, Cauliflower 94.0); Thrombus AUC = 0.935, Sensitivity 93.4%, Specificity 92.6%; TRS R^2^ = 0.98, r = 0.99.	Fusion of morphological and textural features yields near-perfect agreement with clinical outcomes; TRS enables continuous, patient-specific stroke-risk quantification beyond categorical scores.

## Data Availability

The data are available upon request to the corresponding author subramanianjayakrishnan@gmail.com.

## Supplementary Materials

The following supporting information can be downloaded at https://www.mdpi.com/article/10.3390/diagnostics16193154/s1, Table S1: Architecture specification of LAATF, TransUNet, SegFormer, XGBoost, and Clinical TRI-score models; Table S2: Adaptive CLAHE parameter configuration; Table S3: Chan–Vese segmentation implementation details; Table S4: AgileFormer framework configuration and training settings.

## Nomenclature

AF	Atrial Fibrillation
CHA_2_DS_2_-VASc	Clinical Stroke Risk Score
CLAHE	Contrast-Limited Adaptive Histogram Equalization
LAA	Left Atrial Appendage
LAAEV	Left Atrial Appendage Emptying Velocity
LAATF	Left Atrial Appendage Thrombus Framework
LightGBM	Light Gradient Boosting Machine
TEE	Transoesophageal Echocardiography
TRI	Thromboembolic Risk Index
WSS	Wall Shear Stress
