# Supplementary material for: Echocardiographic Framework for Left Atrial Appendage Morphology Assessment and Thromboembolic Risk Stratification in Atrial Fibrillation
Source: Diagnostics (Basel). 2026 Sep 28;16(19):3154. doi: 10.3390/diagnostics16193154 (PMC13664605; doi:10.3390/diagnostics16193154)
Supplement: Supplementary file 1 [file diagnostics-16-03154-s001.zip › diagnostics-4261683-supplementary.pdf]

Table S1: Architecture specification of LAATF, TransUNet, SegFormer, XGBoost, and Clinical TRI-score models

**Model Name**	**Architecture**	**Training Set**	**Augmentation Details**	**Optimizer**	**Learning Rate**	**Batch Size**	**Epochs**	**Loss Function**	**Thresholding**	**Cross-Validation**	**Purpose**
**LAATF (Ours)**	Transformer + U-Net (custom)	192 AF patients (class-balanced: 1,080 images/class)	Rotation ±15°, Scaling 0.9–1.1×, Horizontal flip 50%, Translation ±10%, Brightness ±20%, Contrast ±20%, Gaussian noise σ=0.01	AdamW	1e-4	16	100 (early stopping patience=15)	Dice + Cross-Entropy (weighted for class imbalance)	Argmax (morphology); Youden's J optimised on validation set (thrombus)	Patient-level, nested 5-fold	Primary model (morphology + thrombus + TRI score)
**TransUNet**	Transformer encoder + U-Net decoder (original)	192 AF patients (class-balanced: 1,080 images/class, same folds as LAATF)	Rotation ±15°, Scaling 0.9–1.1×, Horizontal flip 50%, Translation ±10%, Brightness ±20%, Contrast	AdamW	1e-4	16	100 (early stopping patience=15)	Dice + Cross-Entropy (weighted for class imbalance)	Argmax (morphology); Youden's J optimised on validation set (thrombus)	Patient-level, nested 5-fold (same folds as LAATF)	Baseline segmentation model (morphology + thrombus)

			±20%, Gaussian noise σ=0.01								
**SegFormer**	MIT encoder + MLP decoder (B2)	192 AF patients (class-balanced: 1,080 images/class, same folds as LAATF)	Rotation ±15°, Scaling 0.9–1.1×, Horizontal flip 50%, Translation ±10%, Brightness ±20%, Contrast ±20%, Gaussian noise σ=0.01	AdamW	3e-4	16	100 (early stopping patience=15)	Dice + Cross-Entropy (weighted for class imbalance)	Argmax (morphology); Youden's J optimised on validation set (thrombus)	Patient-level, nested 5-fold (same folds as LAATF)	Baseline segmentation model (morphology + thrombus)
**XGBoost**	Gradient boosting (tabular)	192 AF patients (patient-level features extracted from TEE)	None (tabular data; no image augmentation)	N/A (gradient boosting internals)	Learning rate=0.1, max depth=6	N/A	1,000 trees (early stopping patience=50)	Logistic loss (binary classification); Squared error (regression for TRI)	Default 0.5 (classification); optimised threshold for TRI	Patient-level, nested 5-fold (same folds as LAATF)	TRI risk score baseline (tabular)
**Clinical TRI-**	Human cardiology visua	Not applicable (expert	Not applicable (human	Not applicable	Not applicable	Not applicable	Not applicable	Not applicable	Expert binary decision (thro	Not applicable (full	Baseline (morphology +

**Score**	1 assessment	assessment on full test set)	assessment, no augmentation)						mbus); Direct visual classification (morphology)	test set assessed by experts)	thrombus by cardiology experts)

Table S2: Adaptive CLAHE parameter configuration

Category	Parameter	Value	Search Range	Selection Method	Runtime
**Core Parameters**	Tile grid size	8×8	[4×4, 8×8, 12×12, 16×16]	Grid search maximizing entropy	~0.15s/image (CPU)
	Clip limit	2.0–4.0 (adaptive)	[2.0, 2.5, 3.0, 3.5, 4.0]	Optimized per image via quality score	—
**Optimization**	Configurations tested	24	4 clip limits × 6 tile sizes	Exhaustive grid search	~3.6s/image (total search)
**Quality Metrics**	Entropy weight (w_1)	0.30	[0.2, 0.3, 0.4, 0.5]	Fixed from validation	—
	Tenengrad weight (w_2)	0.30	[0.2, 0.3, 0.4, 0.5]	Fixed from validation	—
	RMS contrast weight (w_3)	0.25	[0.15, 0.25, 0.35, 0.45]	Fixed from validation	—
	Noise estimate weight (w_4)	0.15	[0.05, 0.15, 0.25, 0.35]	Fixed from validation	—

Table S3: Chan–Vese segmentation implementation details

Parameter	Selected Value	Search Range	Selection Method	Runtime
Regularization weight (μ)	0.5	0.1, 0.5, 1.0, 2.0	Grid search maximizing Dice score	~0.8 s/image (CPU)
Inside region weight (λ_1)	1.0	0.5, 1.0, 1.5, 2.0	Default balanced setting	—
Outside region weight (λ_2)	1.0	0.5, 1.0, 1.5, 2.0	Default balanced setting	—
Boundary smoothness coefficient (α)	0.3	0.1, 0.3, 0.5, 0.7	Grid search minimizing contour curvature	—
Initial contour generation	Otsu + Adaptive Local Hybrid	—	Fixed initialization protocol	~0.05 s/image
Global threshold (τglobal)	Otsu (automatic)	—	Automatically determined	—
Local threshold (τlocal)	Mean of 3 × 3 neighborhood	—	Automatically determined	—
Maximum iterations	500	—	Grid search with validation	—
Convergence tolerance	1×10^{-4}	1×10^{-3}, 1×10^{-4}, 1×10^{-5}	Fixed for stable convergence	—
Early stopping criterion	ΔEnergy $< 1 \times 10^{-4}$ for 20 consecutive iterations	—	Prevent over-segmentation	~30% runtime reduction
Time step (Δt)	0.1	0.05, 0.1, 0.2	Stability constraint	—
Structuring element	5 × 5 square	3 × 3, 5 × 5, 7 × 7	Selected for optimal LAA morphology	~0.02 s/image
Morphological opening kernel	5 × 5 square	—	Remove small isolated noise	—
Morphological closing kernel	5 × 5 square	—	Fill small holes	—
Morphological dilation kernel	5 × 5 square	—	Connect disjoint segments	—
Total configurations evaluated	80	4 μ × 4 λ × 5 α	Exhaustive grid search	~64 s/validation set
Validation metric	Dice similarity coefficient	—	Maximization objective	—
Optimal configuration	$\mu = 0.5$, $\lambda_1 = 1.0$, $\lambda_2 = 1.0$, $\alpha = 0.3$	—	Dice score = 0.89	—

.

Table S4: AgileFormer framework configuration and training settings.

Category	Parameter	Value	Search Range	Selection Method	Runtime
**Framework**	Deep learning framework	PyTorch v1.9	—	—	—
	Architecture	AgileFormer (custom dual-stream)	—	—	—
**Input**	Input size	384×384×3 (dual stream)	[224×224, 384×384, 512×512]	Grid search optimizing accuracy	~0.01s/image
	Input streams	Contour + Enhanced image	—	Fixed (dual-stream)	—
	Patch size	4×4	—	Fixed (architecture design)	—
**Optimizer**	Optimizer	AdamW	[Adam, AdamW, SGD]	AdamW best for transformers	—
	Learning rate (base)	1e-4	[1e-5, 5e-5, 1e-4, 5e-4, 1e-3]	Grid search (macro F1)	—
	LR scheduler	Cosine annealing	[StepLR, CosineAnnealing, ReduceLROnPlateau]	Smooth convergence	—
	T_max (scheduler)	100 epochs		Match total epochs	—
	Warmup epochs	5		Stabilize early training	—
	Weight decay	0.01	[0.001, 0.01, 0.1]	Grid search (validation loss)	—
	Gradient clipping	1.0 (max norm)	[0.5, 1.0, 2.0, 5.0]	Prevent exploding gradients	—
**Training**	Batch size	16		VRAM constraint + performance	—
	Gradient accumulation	2 steps		Effective batch size = 32	—

	Effective batch size	32	—	16 × 2 accumulation	—
	Epochs	100		Grid search + early stopping	~3.5h/epoch (GPU)
	Early stopping	Patience=15, monitor val loss		Prevent overfitting	Saves ~20% time
	Loss function	Dice + Cross-Entropy (weighted)	—	Handle class imbalance	—
	Class weights	[1.0, 1.2, 1.4, 1.6]	Inverse frequency	Computed from training set	—
